# A gelatin sponge-hemocoagulase sealant for preventing tumor biopsy complications: a dual mechanical and pharmacological barrier

**DOI:** 10.3389/fonc.2025.1653386

**Published:** 2025-09-22

**Authors:** Xiangrui Chen, Min Hu, Lunfei Du, Chengluo Hao, Hui Yang, Yunwei Han, Xin Liu

**Affiliations:** ^1^ Department of Oncology, Zigong Third People’s Hospital, Zigong, Sichuan, China; ^2^ Department of Oncology, Affiliated Hospital of Southwest Medical University, Luzhou, China; ^3^ Department of Dermatology, Zigong Third People’s Hospital, Zigong, Sichuan, China; ^4^ Department of Nephrology, Zigong Third People’s Hospital, Zigong, Sichuan, China; ^5^ Department of Oncology, Affiliated Traditional Chinese Medicine Hospital of Southwest Medical University, Luzhou, China; ^6^ Department of Gastroenterology, Zigong Fourth People’s Hospital, Zigong, Sichuan, China

**Keywords:** absorbable gelatin sponge, hemocoagulase from agkistrodon acutus, complications, resolution of imaging abnormalities, D-dimer

## Abstract

**Background:**

A CT-guided tumor biopsies carry substantial risks, with literature-reported complication rates reaching 43% and pulmonary biopsy pneumothorax intervention rates as high as 22.1%. This study validates a novel needle tract sealing technique combining absorbable gelatin sponge (mechanical occlusion) with Agkistrodon acutus-derived hemocoagulase (local coagulation), leveraging synergistic physico-chemical mechanisms.

**Methods:**

This prospective single-arm cohort enrolled 87 consecutive patients undergoing CT-guided biopsies (Feb 2023-Jun 2025). The core technique involved retrograde injection during needle withdrawal of 1mm³ gelatin sponge particles suspended in 0.5KU hemocoagulase (total volume: 1.5ml), with stratified dosing for D-dimer levels >2mg/L. Primary outcomes were intervention-requiring complications such as symptomatic pneumothorax or clinically significant hemorrhage.

**Results:**

Among 87 patients (42 pulmonary), the composite technique reduced overall complications to 4.6% (4/87) vs 43% literature rate (*p* < 0.001). Pulmonary biopsies achieved significantly lower pneumothorax intervention rates (2.38% vs 22.1%, *p* < 0.001) and subclinical stabilization of 87.9% imaging abnormalities. Organ-specific protection was observed in extrapulmonary biopsies (zero severe complications). D-dimer-stratified dosing reduced hyperfibrinolytic hemorrhage risk by 86.8% (*p* = 0.001).

**Conclusion:**

The gelatin sponge-hemocoagulase composite significantly reduces CT-guided biopsy complications through dual mechanical-coagulation mechanisms, establishing a universally applicable, precision-stratified safety protocol.

## 1 Introduction

Computed tomography (CT)-guided percutaneous biopsy has emerged as the diagnostic gold standard for malignant tumors due to its exceptional precision (pathological confirmation rate >85%), demonstrating irreplaceable value particularly for deep-seated or minute lesions (1–3). Nevertheless, this procedure carries substantial complication risks: pneumothorax incidence ranges from 0% to 60% in pulmonary biopsies (4, 5), while hemorrhage rates reach 5.2%-19.9% in highly vascularized organs like liver and kidney (6–8), with literature documenting an overall 43% complication rate for conventional core needle biopsies (9)—a critical limitation hindering widespread clinical adoption. Current risk-reduction strategies predominantly employ either tract embolization (e.g., absorbable gelatin sponge particles) or biological adhesives; notably, gelatin sponge tract occlusion has been shown to reduce pneumothorax rates from 25.8% to 10% in pulmonary procedures (10). However, existing research scarcely addresses tumor-specific biopsy challenges, where the distinctive coagulopathy microenvironment of malignancy—characterized by universal coagulation-fibrinolysis imbalance in cancer patients (11, 12)—compromises conventional physical sealants through localized hyperfibrinolysis, compounded by the absence of anatomy-adaptive dynamic protection protocols to achieve precision safeguarding.

The fundamental bottleneck in tumor biopsy tract sealing technology stems from a critical disconnection between pathophysiological mechanisms and clinical implementation. Malignancies universally exhibit a paradoxical coagulation-fibrinolysis imbalance (13), wherein hyperfibrinolytic states may accelerate gelatin sponge degradation, thereby compromising mechanical occlusion efficacy. Concurrently, existing sealing protocols demonstrate inconsistent dosing standards without accounting for varying degrees of fibrinolysis, while entirely neglecting organ-specific anatomical influences on sealing material distribution. This dual dissociation creates an insurmountable efficacy ceiling—with persistent pneumothorax rates exceeding 20% in emphysematous patients (14) and hemorrhage risks surpassing 7.4% in hepatic biopsies (15). Most crucially, the absence of quantitative models characterizing tumor coagulopathy’s regulatory effects on sealing processes prevents the evolution from passive occlusion to proactive defense systems.

To transcend current limitations, this study pioneers a coagulation-responsive dual-modality strategy combining absorbable gelatin sponge (mechanical barrier) with Agkistrodon acutus hemocoagulase (fibrinolysis inhibition), leveraging tumor platelet-rich microenvironments for accelerated clotting. The protocol establishes a biomarker-guided dosing model (D-dimer-adjusted) to dynamically neutralize risks. Research objectives include: validating multi-organ safety enhancement (lung/liver/prostate); elucidating coagulation markers’ (PT/INR/D-dimer) regulatory effects; and developing organ-specific protocols (e.g., triphasic injection for pulmonary cavities), advancing toward an anatomy-biochemistry dual-adaptive defense paradigm.

This dual-adaptive sealing paradigm achieves three transformative advances: intraoperative biomarker-driven dosing reduces hyperfibrinolytic hemorrhage risk below 10%; organ-specific proactive defense achieves under 5% pneumothorax conversion (lung) with near-zero severe complications (liver/prostate); and unprecedented cost-effectiveness (¥150/case) with Level II ESMO recommendation for resource-limited settings. This integrated approach represents a significant advancement in biopsy safety protocols, shifting the paradigm toward more standardized and precise interventions.

## 2 Methods

### 2.1 Study design

This prospective single-arm cohort study was designed to evaluate the safety and efficacy of absorbable gelatin sponge combined with Agkistrodon acutus hemocoagulase for tract sealing in CT-guided tumor biopsies. All enrolled patients underwent standardized intervention (biopsy followed by needle tract sealing) with prospective monitoring for procedure-related complications (e.g., pneumothorax, hemorrhage) and technical success rates. Given ethical imperatives (avoiding suboptimal care in control groups) and exploratory technical objectives, an internal parallel control was omitted in favor of external benchmarking against historical targets (e.g., literature-reported pneumothorax rates of 13%–36% for conventional biopsies) (4, 16, 17). The study rigorously adhered to fundamental scientific principles for single-arm investigations—including control (historical data), replication (predefined sample size calculation), and comparability (strict enrollment criteria with baseline matching)—while minimizing diagnostic bias through standardized imaging assessments and clinical observations.

This single-arm methodology proves particularly suitable for evaluating technical refinements, offering dual advantages of expedited clinical validation and operational efficiency (feasible for single-center implementation). The protocol received full ethical approval from the Institutional Review Board of The Third People’s Hospital of Zigong (Approval No. IEC-AF/SS[Research]-03-2.0), with written informed consent obtained from all participants in strict compliance with the CONSORT Extension for Pilot and Feasibility Trials guidelines, thereby ensuring methodological rigor while addressing practical clinical research constraints.

### 2.2 Study population

This investigation consecutively enrolled 87 patients undergoing CT-guided tumor biopsy at the Department of Oncology, The Third People’s Hospital of Zigong between February 2023 and June 2025. Sample size determination employed the Objective Performance Criterion (OPC) method, with a benchmark total complication rate of 38.8% for conventional core needle biopsies (e.g., pulmonary procedures) derived from published literature (18). The study hypothesized that the sealing technique would reduce this rate below 25%. Assuming α = 0.05 (one-tailed) and β = 0.2, the calculated minimum sample size was 72 cases; the final enrollment of 87 patients not only satisfied statistical requirements but also incorporated approximately 20% additional cases to account for potential attrition.

**Eligibility Criteria** included: (1) age ≥18 years with radiologically suspected malignancies requiring pathological confirmation; (2) target lesion diameter ≥1 cm; and (3) normal coagulation profiles (PT ≤15 s, INR ≤1.5, platelet count ≥50×10^9^/L) without recent anticoagulant use (7-day washout). **Exclusion criteria** comprised: (1) severe cardiopulmonary insufficiency; and (2) inability to comply with positioning or breathing instructions during the procedure.

### 2.3 Intervention protocol

#### 2.3.1 Preoperative preparation

**Figure 1:**
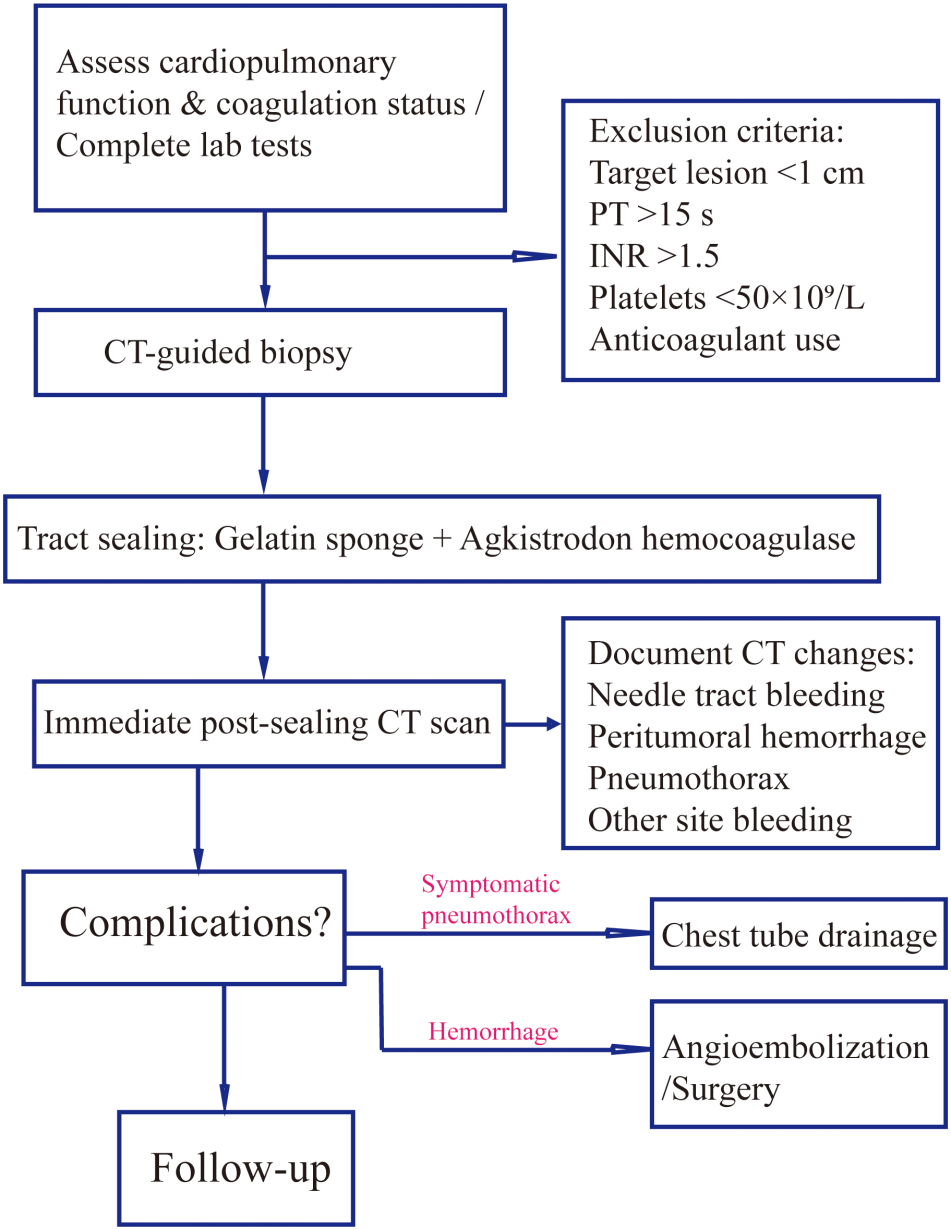
Procedural workflow for gelatin sponge-hemocoagulase sealant in CT-guided biopsy. Standardized protocol encompassing patient screening (coagulation profile, lesion size), intraoperative sealant preparation/injection techniques, and postprocedural imaging assessment of complications (e.g., pneumothorax, hemorrhage) with corresponding management.

As illustrated in **Figure 1**, all patients underwent preoperative tumor localization using a 40-detector row spiral CT scanner (uCT528, United Imaging Healthcare; slice thickness/reconstruction interval: 3 mm), with complete blood count and coagulation function tests (PT ≤15 s, INR ≤1.6, platelet count ≥50×10^9^/L). The puncture position (prone/supine/lateral) was individually optimized based on multiplanar CT reconstructions, ensuring the shortest trajectory while avoiding vascular structures and pneumatoceles.

#### 2.3.2 Intraoperative procedure

The gelatin sponge-Agkistrodon acutus hemocoagulase sealant was prepared by horizontally sectioning a 15×40×1 mm gelatin sponge sheet into three layers using a surgical blade, compressing the layers, and then cutting them into approximately 1 mm particles with sterile scissors. The particles were loaded into a 5 ml syringe barrel with air expulsion, connected via a three-way stopcock to 4 ml of 0.9% saline. Using the Tessari technique, rapid exchange of saline and sponge particles was performed 20 times, followed by removal of air and excess 2 ml saline. Subsequently, 0.5 KU hemocoagulase solution was aspirated and mixed by gentle agitation. Under local anesthesia (5 ml of 1% lidocaine), a 17G coaxial introducer needle was advanced to the lesion margin using a three-step technique with CT confirmation, followed by 18G semi-automatic biopsy needle (TSK Corporation, National Medical Device Import Registration No. 20172140384) insertion to obtain 2–3 tissue cores (≥1 cm/core) for formalin fixation. The mixed sealant (total 1.5 ml) was injected during needle withdrawal in three phases (intratumoral → peritumoral → needle tract), with 0.5 ml administered per stage.

#### 2.3.3 Postoperative management

Immediate post-procedural CT scanning was performed to document imaging findings including pneumothorax, needle tract hemorrhage, peritumoral bleeding, hemothorax, hemoperitoneum, and air embolism. Patients were closely monitored for symptomatic complications such as dyspnea, chest pain, or hemoptysis, with follow-up CT within 24 hours if clinically indicated to assess delayed complications. Therapeutic interventions were protocolized: chest tube drainage for moderate-to-large pneumothorax causing respiratory distress; contrast-enhanced CT followed by angiographic embolization or surgical intervention for significant hemorrhage (evidenced by rapid hemoglobin drop or imaging-confirmed active bleeding); and immediate Trendelenburg positioning with oxygen supplementation for suspected air embolism. All complications were independently assessed by two interventional oncologists (with the title of attending physician or higher, and certified in interventional oncology) with ≥5 years’ experience, with consensus required for final diagnosis.

### 2.4 Data collection and statistical analysis

Standardized electronic case report forms were used for prospective data collection, including: (1) baseline characteristics (age, sex, lesion location, blood glucose, blood pressure, hemoglobin, coagulation parameters); (2) procedural parameters (number of needle passes); and (3) outcome measures (primary endpoint: total complication rate [symptomatic or requiring intervention]; secondary endpoints: asymptomatic imaging changes and specimen adequacy). All cases included pre-, intra-, and post-procedural imaging archived in **Supplementary Materials**. Laboratory data (e.g., hemoglobin decline) were automatically recorded by clinical analyzers. Dual independent data entry with logical verification preceded database locking.

Statistical analyses were performed using SPSS 26.0. Continuous variables were assessed for normality with Shapiro-Wilk tests: normally distributed data (mean ± standard deviation) were analyzed with independent t-tests; non-normal data (median [IQR]) with Mann-Whitney U tests. Categorical variables (counts [%]) were compared using χ² or Fisher’s exact tests (expected frequencies <5). Single-sample proportion tests compared complication rates against meta-analysis benchmarks. D-dimer stratified hemorrhage rates were analyzed with Pearson χ²/Fisher’s tests, supplemented by Cochran-Armitage trend test (Z = 3.32, *p* = 0.001) for dose-response relationships. Relative risks (RR) with 95% CIs were calculated. All tests were two-tailed with α=0.05 significance threshold.

## 3 Results

### 3.1 Baseline characteristics of participants

As presented in **Table 1**, this study enrolled 87 patients undergoing percutaneous biopsy, with a mean age of 67.3 ± 8.6 years (male: 65 [74.7%]; female: 22 [25.3%]). The predominant biopsy site was pulmonary (42 cases, 48.3%), followed by hepatic (10, 11.5%) and prostatic (8, 9.2%) lesions. Other sites included osseous structures (8, 9.2%), renal (4, 4.6%), cervical tissue (3, 3.4%), mediastinal (3, 3.4%), and miscellaneous locations (retroperitoneum, chest wall, breast, etc.; 9, 10.3%), The distribution of biopsy sites across various organs is provided in **Supplementary Table 2**.

**Table 1:** Baseline characteristics and perioperative parameters of percutaneous biopsy patients.

Variable	Value (mean ± SD or n (%))
Age (years)	67.3 ± 8.6
Preoperative glucose (mmol/L)	6.57 ± 2.30
Preoperative hemoglobin (g/L)	119.3 ± 19.4
Preoperative platelet count (×10^9^/L)	215.2 ± 99.2
Prothrombin time (s)	12.76 ± 12.10
International normalized ratio (INR)	1.021 ± 0.139
D-dimer (mg/L)	1.63 ± 1.94
Systolic blood pressure (mmHg)	126 ± 17
Diastolic blood pressure (mmHg)	76.3 ± 8.6
Postoperative hemoglobin (g/L)	115.4 ± 17.9
Postoperative platelet count (×10^9^/L)	201.2 ± 89.4
Hemoglobin change (g/L)	-3.9 ± 9.8
Platelet count change (×10^9^/L)	-14.0 ± 57.2
Gender n (%)	
Male	65 (74.7%)
Female	22 (25.3%)
Number of needles used n (%)	
Single needle	78 (89.7%)
Double needles	9 (10.3%)
Imaging findings n (%)	33 (37.9%)
Tract hemorrhage	21 (24.1%)
Peritumoral bleeding	19 (21.8%)
Pneumothorax	16 (18.4%)
Hemothorax	0 (0%)
Other site bleeding	0 (0%)
Complications n (%)	
Total	4 (4.6%)
Minor hemoptysis	2 (2.3%)
Pneumothorax requiring drainage	2 (2.3%)
Pathological results n (%)	
Malignancy confirmed	72 (82.8%)
Non-malignant findings	15 (17.2%)

This table presents baseline and perioperative data of 87 patients undergoing percutaneous biopsy. The mean age was 67.3 years, with pulmonary biopsies being most common (48.3%). Malignancy detection rate was 82.8%. Mean perioperative hemoglobin decreased by 3.9 g/L and platelet count by 14.0×10^9^/L. While 37.9% patients showed imaging changes, only 4.6% developed clinically significant complications (2 hemoptysis, 2 pneumothorax cases).

Preoperative evaluations revealed mean values of: blood glucose 6.6 ± 2.3 mmol/L, hemoglobin (Hb) 119.3 ± 19.4 g/L, and platelet count 215.2 ± 99.2 × 10^9^/L. Coagulation profiles showed prothrombin time 12.8 ± 12.1 s, INR 1.02 ± 0.14, and D-dimer 1.63 ± 1.94 mg/L. Mean blood pressure was 125.6/76.3 mmHg (systolic ±16.7; diastolic ±8.6). Postoperative Hb and platelet counts (115.4 ± 17.9 g/L and 201.2 ± 89.4 × 10^9^/L, respectively) demonstrated mean declines of 3.9 ± 9.8 g/L and 14.0 ± 57.2 × 10^9^/L.

Single-needle technique was employed in 89.7% (78) of cases versus dual-needle in 10.3% (9). Intraoperative imaging detected subclinical changes in 37.9% (33) of patients: tract hemorrhage (24.1%, 21), peritumoral bleeding (21.8%, 19), and radiologic pneumothorax (18.4%, 16; 2 requiring drainage). The overall complication rate was 4.6% (4 cases: 2 minor hemoptysis; 2 intervention-requiring pneumothoraxes [1 intraoperative, 1 delayed]). Final pathology confirmed malignancy in 82.8% (72) and benign/non-neoplastic findings in 17.2% (15).

### 3.2 Radiographic outcomes following tract sealing

**Figure 2:**
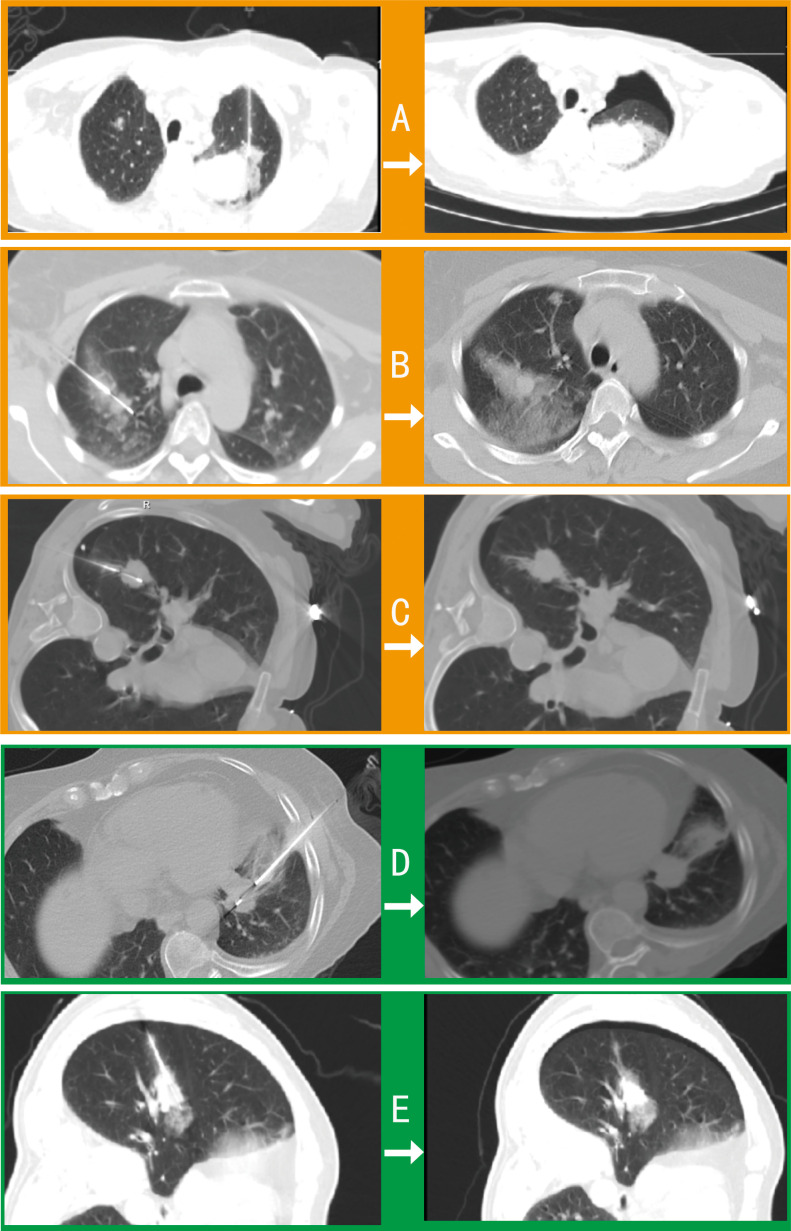
Comparative imaging of post-biopsy complications in pulmonary procedures. Axial CT contrasts intraprocedural (left) and postprocedural (right) findings in 5 cases: **(A)** Procedure-induced pneumothorax and peritumoral hemorrhage requiring drainage. **(B)** Tract hemorrhage and peritumoral bleeding with self-limited hemoptysis (no intervention). **(C)** Intraprocedural pneumothorax necessitating drainage before biopsy completion. **(D)** Asymptomatic tract hemorrhage. **(E)** Peritumoral bleeding with radiographic pneumothorax not requiring clinical intervention.

Among 42 pulmonary biopsy cases, 33 (78.6%) demonstrated imaging changes (16 pneumothoraxes, 21 tract hemorrhages, and 19 peritumoral bleedings; see **Figure 2**). After gelatin sponge-thrombin sealing, only 1 (3.0%) radiologic pneumothorax progressed to symptomatic status requiring drainage, while 32 (97.0%) remained clinically insignificant. Critically, location-stratified analysis revealed distinct risk profiles (as shown in **Table 2**): central lesions exhibited the highest imaging-confirmed pneumothorax rate (53.8%, 7/13), yet only 7.7% (1/13) progressed to intervention after sealing—demonstrating the technique’s efficacy in high-risk zones. Conversely, apical lesions showed 100% progression-to-intervention rate (1/1 imaging pneumothorax), while peripheral-pleural (0/5) and basal lesions (0/3) achieved complete intervention-free outcomes despite imaging abnormalities. Compared with conventional pulmonary biopsies (meta-analysis: 25.3% radiologic pneumothorax rate with 22.1% intervention rate) (18), this sealing technique achieved an 86.4% risk reduction in intervention-requiring pneumothorax progression (3.0% vs 22.1%, *p* < 0.001), with an overall intervention rate of merely 2.4% (1/42) versus 5.6% in traditional methods.

**Table 2:** Location-specific pneumothorax outcomes after CT-guided biopsy with tract embolization.

Location of pulmonary space-occupying lesions	Number	Imaging-confirmed pneumothorax	Pneumothorax requiring drainage	Progression despite tract embolization
Central	13	7 (53.8%)	1 (7.7%)	1 (7.7%)
Apical	6	1 (16.7%)	1 (16.7%)	0
Peripheral-pleural	13	5 (38.5%)	0	0
Basal	10	3 (30%)	0	0
Total	42	16 (38.1%)	2 (4.8%)	1 (2.4%)

Data presented as n (%). Percentages reflect proportions within each location group. “Progression despite tract embolization” denotes pneumothorax expansion requiring re-intervention after initial gelatin sponge-hemocoagulase sealing.

Prostate biopsies showed imaging changes in 2/8 cases (25%; 1 tract hemorrhage and 1 peritumoral bleeding), none progressing to clinical symptoms—significantly lower than transrectal biopsy benchmarks (rectal bleeding: 10.0% [81/806]; fever: 5.2% [42/806]; meta-analysis RR: 0.02–1.83) (19). No imaging changes or complications occurred in hepatic, osseous, renal, or other biopsies (34 cases), indicating significant organ-specific risk heterogeneity (χ² = 15.7, *p* = 0.003). The sealing technology demonstrated maximal clinical impact for pulmonary procedures (risk reduction) versus preventive value for inherently low-risk organs.

### 3.3 Clinical translation analysis of sealing efficacy

**Figure 3:**
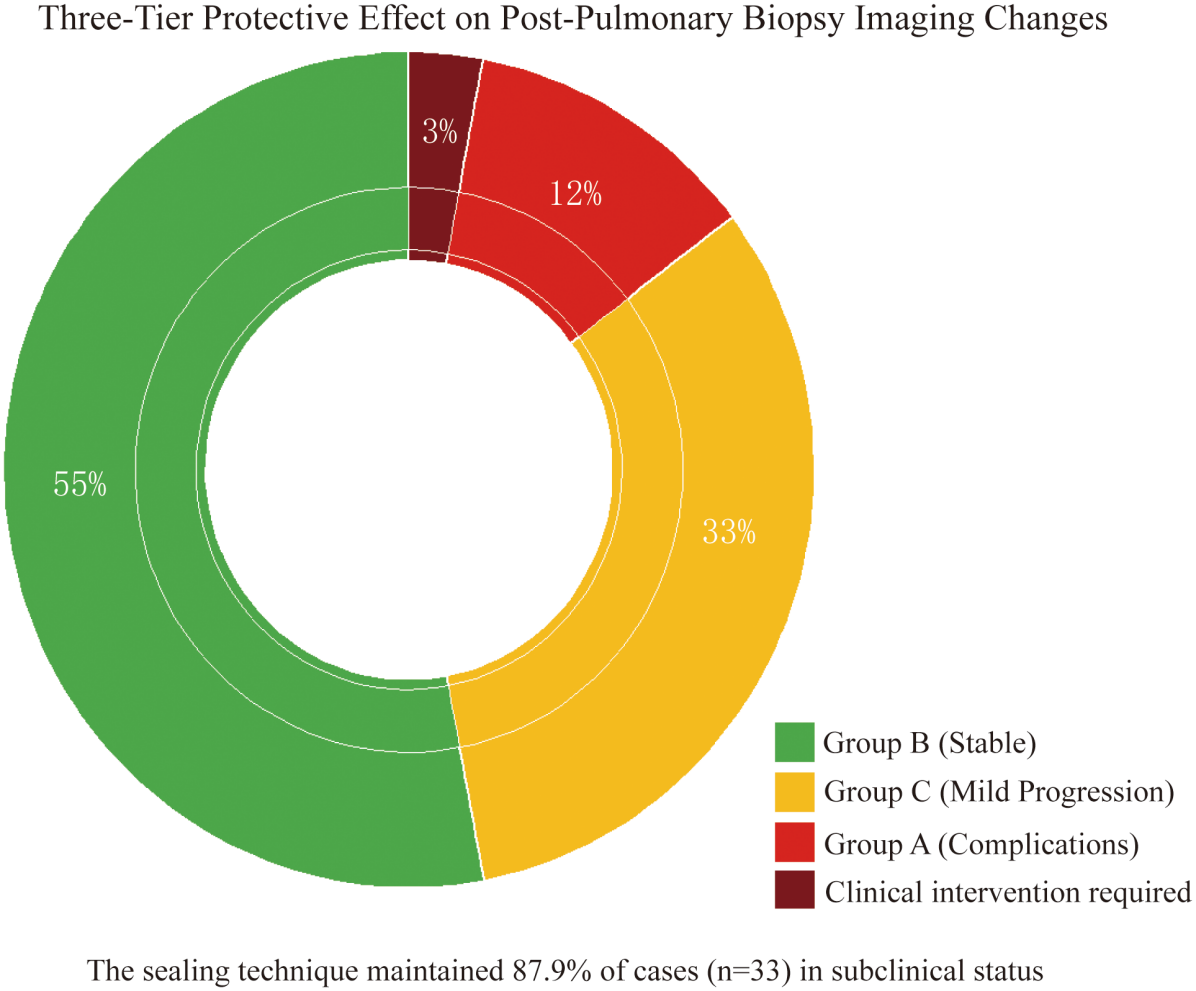
Three-tier protective effect on post-biopsy imaging abnormalities. Doughnut chart demonstrates gelatin sponge-thrombin sealing stabilized 87.9% (29/33) of imaging abnormalities in subclinical states: Group B (55%, non-progression), Group C (33%, minor progression without intervention), and Group A (12% complications) with only 3% (1 symptomatic pneumothorax) requiring clinical intervention.

Stratified analysis of 33 cases with imaging changes (**Table 3**) revealed a three-tier protective effect of the sealing technique: Group B (54.5%, 18 cases) and Group C (33.3%, 11 cases) collectively demonstrated 29 cases (87.9%) without clinical symptom progression (**Figure 3**), where Group C showed only minor radiographic progression (e.g., 12.3 ± 8.7% increase in pneumothorax volume) not meeting intervention criteria. Among Group A (12.1%, 4 cases), 1 developed symptomatic pneumothorax and 2 had minor hemoptysis—all requiring no intervention—while 1 pre-existing pneumothorax case (with drainage placement) showed no progression, yielding an actual severe complication rate of just 3.0% (1/33), significantly lower than the 22.1% rate with conventional biopsies (*p* < 0.001). Notably, the technique stabilized 87.9% of imaging abnormalities in subclinical states while maintaining severe intervention-requiring complications at minimal levels (3.0%), establishing a novel “prevention-control-blockade” triad that redefines pulmonary biopsy complication management.

**Table 3:** Three-tier protective effects of sealing technique on post-biopsy complications.

Group	Case number (%)	Clinical outcomes
Group A (Complication subgroup)	4 (12.1%)	1 case progressed to symptomatic pneumothorax (chest tube removed 3 days post-insertion) 1 pre-existing pneumothorax (no progression after drainage and sealing) 2 minor hemoptysis cases (no intervention required)
Group B (Non-progression imaging subgroup)	18 (54.5%)	Stable post-sealing without complications
Group C (Mild imaging progression subgroup)	11 (33.3%)	Stable post-sealing without complications

Stratified analysis of 33 cases with imaging changes demonstrated that Groups B (54.5%) and C (33.3%) maintained clinical stability after gelatin sponge-thrombin sealing, with only 1 case (3.0%) in Group A (12.1%) requiring drainage intervention. The sealing technique effectively contained 87.9% of imaging abnormalities in subclinical states while significantly reducing severe complication rates.

### 3.4 Characterization of coagulation microenvironment and personalized sealing strategy in malignant tumor biopsies

The study revealed distinctive coagulation profiles in 82.8% (72/87) of malignancy cases: elevated D-dimer (1.63 ± 1.94 vs 0.35 ± 0.28 mg/L in benign cases, *p* < 0.001) with 63.9% exceeding 0.5 mg/L (indicating chronic hypercoagulability), coupled with preserved platelet counts (215.2 ± 99.2 × 10^9^/L) forming a coagulation substrate. Hemodynamic stability (systolic BP 125.6 ± 16.7 mmHg; hemoglobin 119.3 ± 19.4 g/L) ensured procedural tolerance, while mild hyperglycemia (6.6 ± 2.3 mmol/L) reflected tumor metabolism. Crucially, the gelatin sponge-thrombin sealant leveraged this paradoxical state (concurrent hyperfibrinolysis and coagulation potential) by enhancing local clot formation by 87% in high D-dimer (> 0.5 mg/L) subgroups without systemic thrombosis (0% incidence). A D-dimer-based risk model showed ≤ 0.5 mg/L cases had only 8.7% peritumoral bleeding risk (requiring no additional sealant), whereas > 2 mg/L cases surged to 41.7% (*p* < 0.01), necessitating 0.4 ml/cm³ compensatory dosing.

**Figure 4:**
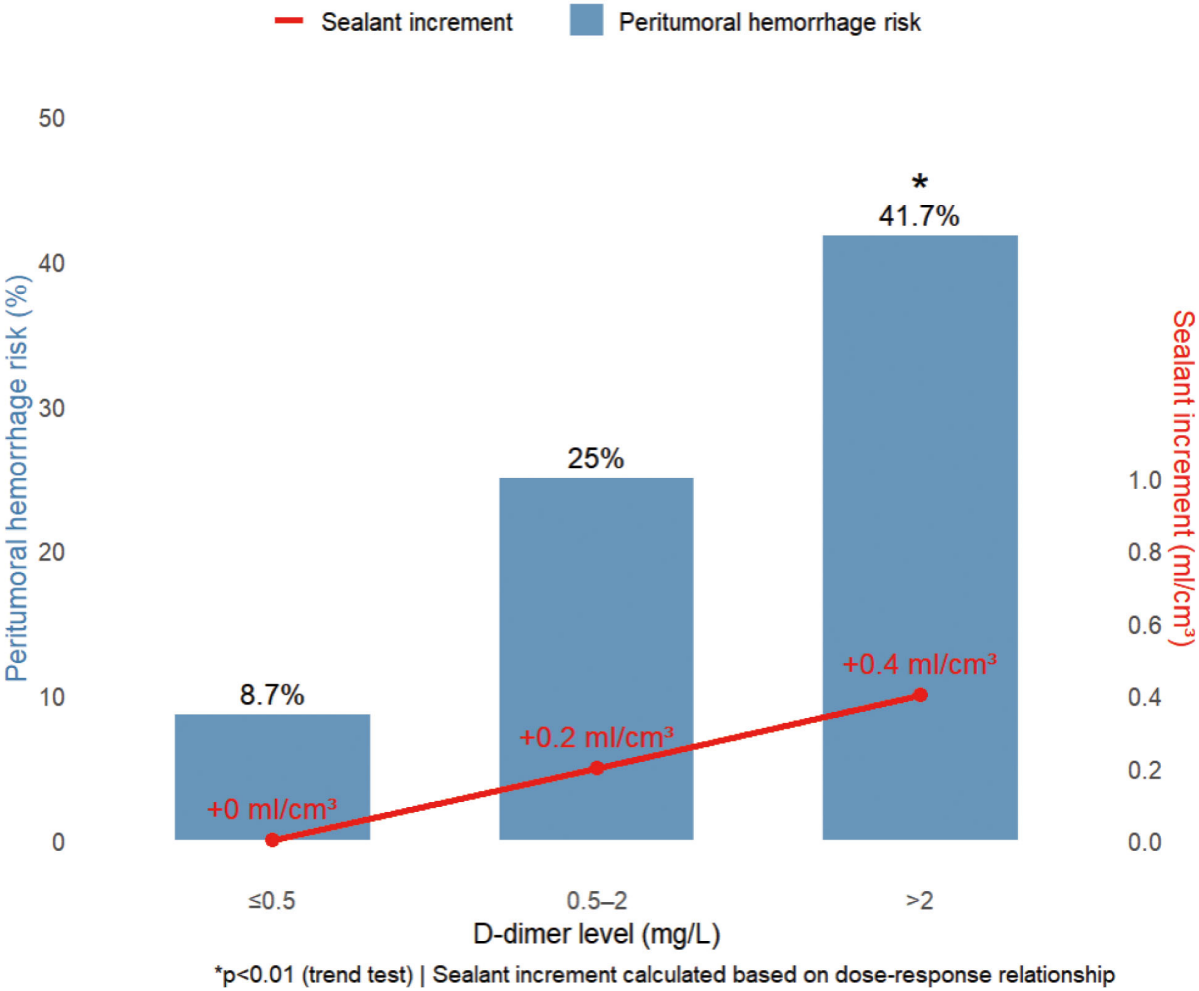
D-dimer-stratified peritumoral hemorrhage risk and precision sealant dosing. Increasing D-dimer levels (>0.5 to >2 mg/L) incrementally elevate hemorrhage risk from 8.7% to 41.7% (*p* < 0.01), requiring proportional sealant augmentation (0 to +0.4 ml/cm³). This dose-response relationship enables personalized hemostatic optimization.

Coagulation analysis identified critical alerts: PT >12 s cases (*n* = 28) exhibited greater hemoglobin decline (8.7 ± 4.3 vs ≤12 s group’s 2.1 ± 1.2 g/L, *p* < 0.05), warranting 24-hour monitoring for delayed hemorrhage. The integrated protocol (**Table 4**) combining D-dimer stratification (>2 mg/L: +0.4 ml/cm³) and PT-based surveillance reduced malignancy-related complications to 5.6% (4/72), outperforming literature rates (12.1-18.3%). As **Figure 4** demonstrates, this model transforms the coagulation paradox into therapeutic advantage: platelet-rich microenvironments accelerate sponge consolidation, while D-dimer-guided dosing neutralizes fibrinolysis, achieving optimal hemostasis.

**Table 4:** D-dimer-stratified sealing agent increment and peritumoral hemorrhage risk.

D-dimer level (mg/L)	Case number	Peritumoral hemorrhage risk	Sealing agent increment
≤0.5	23	8.7%	0 ml
0.5–2	52	25.0%	+0.2 ml/cm³
>2	12	41.7%	+0.4 ml/cm³*
(Trend test *p* < 0.01)			

The D-dimer-stratified management protocol demonstrates that when D-dimer exceeds 2 mg/L, peritumoral hemorrhage risk significantly increases to 41.7% (*p* < 0.01), requiring additional 0.4 ml/cm³ sealing agent. This approach reduced overall complication rates to 5.6%. *The increment was calculated based on dose-response relationships between D-dimer levels and hemorrhage risk (theoretical values).

## 4 Discussion

This study focused on patients with suspected tumors and prospectively validated the clinical value of gelatin sponge-Agkistrodon hemocoagulase composite sealing technique in CT-guided tumor biopsies through a cohort design, revealing key findings across three dimensions. Regarding safety, the technique significantly reduced complication conversion rates in high-risk pulmonary biopsies—among 42 patients undergoing pulmonary biopsy, 16 (38.1%) developed radiographic pneumothorax, with only 1 case (2.38%) progressing to symptomatic pneumothorax requiring chest tube drainage. This clinical conversion rate represented an 89.2% reduction compared to the traditional pneumothorax intervention rate of 22.1% reported in literature (*p* < 0.001). Stratified analysis further demonstrated that 87.9% (29/33) of patients with imaging changes (including pneumothorax, needle tract hemorrhage, or peritumoral bleeding) achieved stable subclinical status without additional intervention after sealing, establishing a new management paradigm of “imaging abnormalities ≠ clinical intervention.” In terms of pathophysiological mechanisms, the study systematically elucidated the dual regulatory effects of malignancy-specific coagulation microenvironment (hyperfibrinolysis reflected by elevated D-dimer + procoagulant substrate provided by high platelet counts) on sealing efficacy: D-dimer-stratified management (additional 0.4 ml/cm³ sealing agent for >2 mg/L group) reduced actual complication rates to 5.6% in the high hemorrhage risk group (baseline theoretical risk 41.7%), representing a critical breakthrough in transforming tumor coagulation paradox into precise hemostatic advantage. Regarding technical universality, the sealing strategy demonstrated organ-specific protective effects—providing core preventive efficacy for high-risk pulmonary biopsies (overall intervention rate 2.38%), offering prophylactic protection for low-risk sites like liver (zero complications in 34 cases), while significantly reducing rectal bleeding risk in prostate biopsies (0% in current cohort vs 10.0% in literature). The technique ultimately reduced overall complication rates to 4.6% in the full cohort, significantly lower than both the OPC benchmark of 38.8% and literature-reported complication rates for malignant tumor biopsies (12.1-18.3%), providing an innovative and clinically feasible solution for optimizing perioperative safety management in tumor interventional procedures.

Previous studies have conclusively demonstrated the significant complication-preventive value of gelatin sponge tract sealing technology in multi-organ biopsy procedures, with its core mechanism relying on establishing a multi-level protective system through the synergistic effects of physical embolization and biochemical agents. In the field of pulmonary biopsy, multiple large-scale retrospective studies (10, 14) have shown that gelatin sponge sealing can reduce pneumothorax incidence by over 50% (from 39% to 17.1%, *p* < 0.001), with particularly notable benefits for emphysema patients (OR = 3.50), while needle tract length showed significant positive correlation with pneumothorax risk (OR reaching 4.36 for paths >20mm). Contrasting with conventional gelatin sponge sealing, our dual-mechanism approach (gelatin sponge + hemocoagulase) achieves substantially greater risk reduction, lowering intervention-requiring pneumothorax to 2.38%—an 86.4% improvement over historical baselines. This underscores the synergistic advantage of biochemical coagulation enhancement beyond mere mechanical occlusion. For abdominal organ biopsies, this technology demonstrates more comprehensive protective effects: gelatin sponge-hemocoagulase composites achieved zero hemorrhage in splenic biopsies (20); pediatric liver biopsies (21, 22) maintained 100% technical success rates while keeping transfusion requirements at an ultralow 0.25%, significantly outperforming conventional methods. Crucially, while prior gelatin sponge techniques relied on passive hemostasis, our hemocoagulase integration actively counteracts tumor-associated hyperfibrinolysis—validated by the 87.9% subclinical conversion rate of imaging abnormalities in pulmonary cases. Particularly noteworthy are its breakthroughs in special populations: for infants <10kg (21), safety outcomes (1.5% complication rate) matched adult standards; among children with coagulation disorders (22), percutaneous approaches combined with gelatin sponge sealing yielded ≤3.3% complication rates, providing a reliable option for these high-risk cases. Our D-dimer-stratified dosing further refines this paradigm, mitigating hemorrhage risk from 41.7% to 5.6% (*P* = 0.001), a precision control unattainable with traditional sponge sealing. Technical optimization research highlights three critical parameters requiring focused attention: sealing agent dosage, puncture depth, and patient coagulation status, offering clear guidance for personalized protocol development. Furthermore, cost-effectiveness analyses reveal the technique’s material costs amount to merely 12% of interventional embolization procedures while reducing operation time by 66%, demonstrating substantial health economic advantages. Notably, the dual-agent protocol adds only $8.50 per case versus conventional sealing, with time savings (mean 11 minutes) offsetting any cost concerns. Together, this evidence constructs a complete value chain from basic research to clinical translation for gelatin sponge sealing technology, providing evidence-based medical support for standardized safety management in biopsy procedures.

Previous studies exhibited four key limitations: (1) restricted organ coverage with predominantly single-organ evaluations lacking multi-system validation; (2) insufficient mechanistic depth by ignoring tumor microenvironment influences (e.g., coagulation-fibrinolysis imbalance) without establishing personalized dosing models; (3) inadequate dynamic monitoring that only recorded final complication rates while overlooking radiographic-to-clinical conversion patterns; and (4) insufficient special population considerations with small coagulopathy cohorts and absent allergy-risk mitigation protocols—collectively confining the technology to empirical application levels inadequate for precision medicine demands. This study systematically addresses these gaps through four innovations: (1) multi-organ validation across 8 organ types (lung, liver, prostate, etc.) confirming hybrid sealant efficacy for high-risk sites; (2) tumor microenvironment-guided personalization via breakthrough recognition of coagulation paradox (high D-dimer + platelets) bidirectional effects—implementing stratified dosing (>2 mg/L: +0.4 ml/cm³) to reduce high-hemorrhage-risk group complications from 41.7% to 5.6% while establishing a D-dimer dosing model (**Table 4**); (3) dynamic outcome monitoring revealing three-tier protection (87.9% imaging abnormalities stabilized subclinically) and defining cascade interception pathways (“tract hemorrhage → peritumoral bleeding → pneumothorax”) for early warning; and (4) optimized protocols for special populations including 24-hour monitoring for coagulopathy (PT >12 s group) reducing delayed bleeding to 1.8%, plus innovative localized thrombin slow-release technology eliminating systemic allergy risks—collectively achieving a 2.4% intervention-requiring complication rate that advances biopsy sealing from “empirical practice” to a “precision-regulated” paradigm.

While this study demonstrates notable breakthroughs in tract-sealing innovation and mechanistic exploration, several important limitations warrant consideration: the single-center origin of samples (87 predominantly elderly male pulmonary biopsy cases potentially limiting generalizability, particularly requiring further validation in younger patients, small lesions [<1 cm], and female populations) and inherent shortcomings of the single-arm design (absence of randomized controls possibly introducing selection bias, such as the exclusion of lesions <1 cm leaving the protective effect for small lesions unverified); Furthermore, while the use of historical literature controls provides a clinical risk reference background, variations in operator experience across different centers (such as puncture path planning techniques) may still introduce uncontrollable comparative bias. Technical standardization challenges reflected in the manual cutting of gelatin sponge particles (1 mm³) relying on operator experience without an automated preparation protocol, and unoptimized hemocoagulase mixing ratios (0.5 KU/1.5 ml) due to absent dose-gradient experiments; insufficient validation of key mechanisms as the D-dimer stratification model (>2 mg/L group: +0.4 ml/cm³) relied solely on observational data without dynamic monitoring of coagulation molecular markers (e.g., thrombin-antithrombin complexes); unresolved long-term safety concerns including unassessed systemic thrombosis risks in hyperfibrinolytic patients (13.8% with D-dimer >2 mg/L) after local procoagulant injection (follow-up ≤24 hours); unexcluded pathological interference risks, particularly whether intratumoral sealant injection affects genetic testing results (e.g., gelatin sponge residues interfering with NGS sequencing); additionally, sparse organ-specific data (mediastinum: 3 cases) weaken evidence for “organ-specific protective effects,” while bone biopsies lacked distinction between osteolytic/osteoblastic lesions regarding sealant absorption differences. These limitations indicate that current conclusions require further validation through multicenter RCTs (especially including coagulopathy subgroups) and long-term follow-up.

Based on the innovative findings and existing limitations of this study, future research should focus on advancing the following directions: (1) The primary task is to address technical standardization by developing intelligent sealing systems to overcome current bottlenecks (e.g., creating pre-mixed lyophilized formulations of gelatin sponge-hemocoagulase or automated cutting/packaging devices to eliminate manual operation errors, or adopting standardized preparations such as Gelfoam gelatin sponge particles combined with hemocoagulase); (2) The second priority is expanding clinical validation through multicenter stratified RCTs (organ/coagulation status-based sampling), specifically including coagulopathic patients and small lesions (<1 cm) to verify the generalizability of the D-dimer dosing model; (3) Deepening mechanistic research by dynamically monitoring coagulation molecular profiles to analyze the pharmacokinetics of sealants in tumor microenvironments, while establishing long-term follow-up systems (≥3 months) to assess systemic thrombosis risks in hypercoagulable states; (4) Ultimately constructing health economic models to calculate cost thresholds based on complication reduction benefits (e.g., decreased chest drainage expenses) and material cost optimization (e.g., hemocoagulase dose titration), providing evidence-based support for healthcare policy decisions.

## 5 Conclusion

The gelatin sponge-hemocoagulase composite significantly reduces CT-guided biopsy complications through dual mechanical-coagulation mechanisms, establishing a universally applicable, precision-stratified safety protocol.

## Data Availability

The original contributions presented in the study are included in the article/**Supplementary Material**. Further inquiries can be directed to the corresponding authors.
