# Supplementary material for: A gelatin sponge-hemocoagulase sealant for preventing tumor biopsy complications: a dual mechanical and pharmacological barrier
Source: Front Oncol. 2025 Sep 22;15:1653386. doi: 10.3389/fonc.2025.1653386 (PMC12497518; doi:10.3389/fonc.2025.1653386)

1. **Supplementary Table 1**. Missing Covariates and Interpolation Methods

| Covariate | Method for Missing Data | Missing Values | Data imputation |
| --- | --- | --- | --- |
| Preoperative Blood Glucose | Median imputation | 5 (5.75%)​ | 6.14 |
| D-Dimer | Median imputation | 10 (11.49%)​ | 0.86 |
| Postoperative Hemoglobin (Hb) | Mean imputation | 19 (21.84%)​ | 119.12 |
| Postoperative Platelet (PLT) | Mean imputation | 19 (21.84%)​ | 186.37 |

**Supplementary Table 1 Note:** Missing data in covariates were primarily due to incomplete laboratory records or logistical constraints during data collection. Median imputation was used for **Preoperative Blood Glucose** and **D-Dimer** to preserve robustness against outliers, while mean imputation was applied to **Postoperative Hemoglobin (Hb)** and **Platelet (PLT)** under the assumption of normally distributed values. These methods were chosen to minimize bias and maintain statistical power, with imputed values reflecting central tendencies of the observed data. Missingness ranged from 5.75% to 21.84%, within acceptable limits for single imputation approaches.

1. **Supplementary Table 2.** Distribution of Percutaneous Biopsy Sites

| Body Part | Count | Percentage |
| --- | --- | --- |
| Lung | 42 | 48.28% |
| Liver | 10 | 11.49% |
| Prostate | 8 | 9.2% |
| Ilium | 4 | 4.6% |
| Kidney | 4 | 4.6% |
| Mediastinum | 4 | 4.6% |
| Neck tissue | 3 | 3.45% |
| Abdominal tissue | 2 | 2.3% |
| Breast tissue | 2 | 2.3% |
| Thoracic vertebrae | 2 | 2.3% |
| Chest wall | 1 | 1.15% |
| Lumbar vertebrae | 1 | 1.15% |
| Pelvic wall | 1 | 1.15% |
| Rectum | 1 | 1.15% |
| Retroperitoneal organs | 1 | 1.15% |
| Sternum | 1 | 1.15% |
| ​Total​ | 87 | 100%​ |

1. **The CT images of each participant (sorted by participant ID, ID 1~40).**
2.
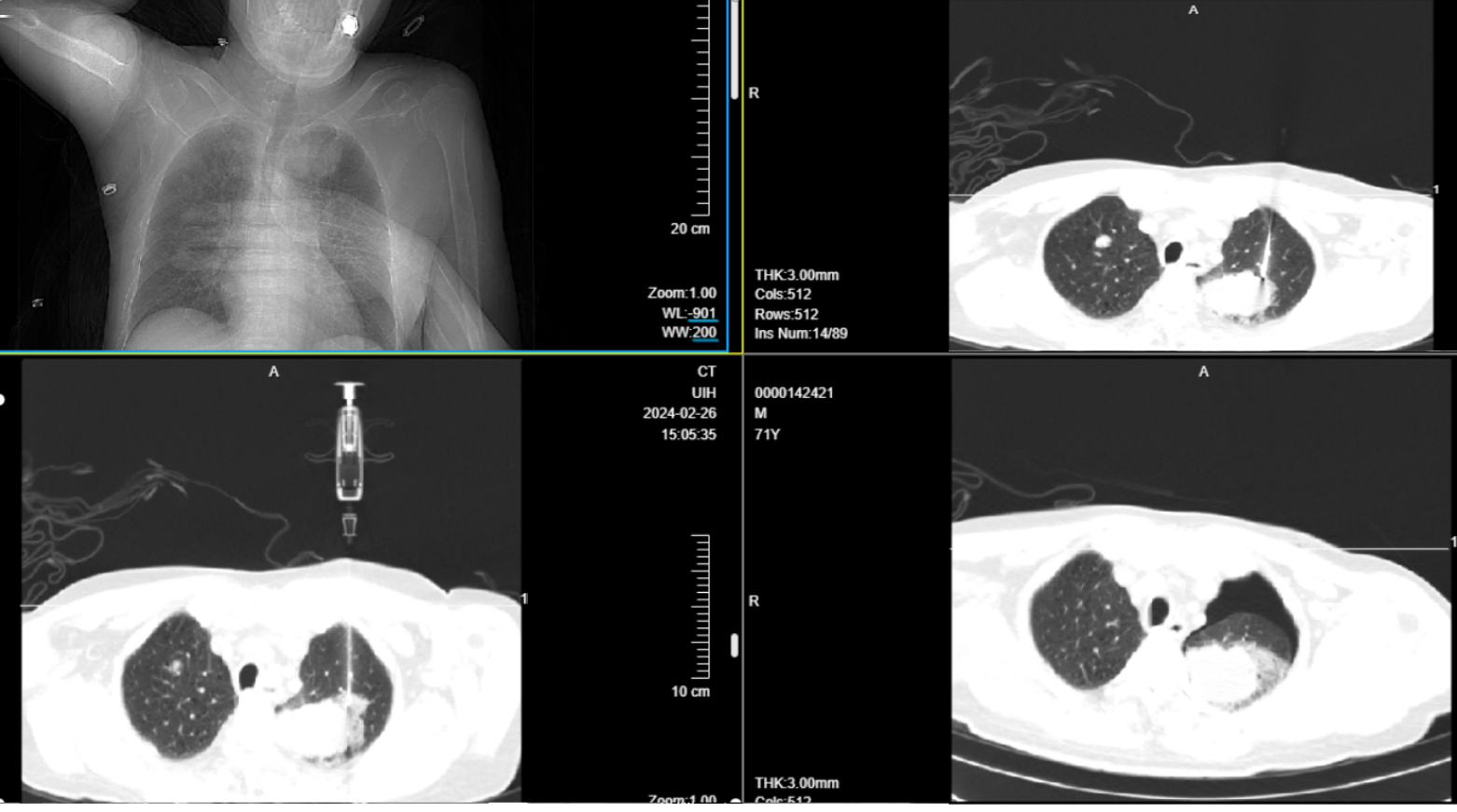

3.
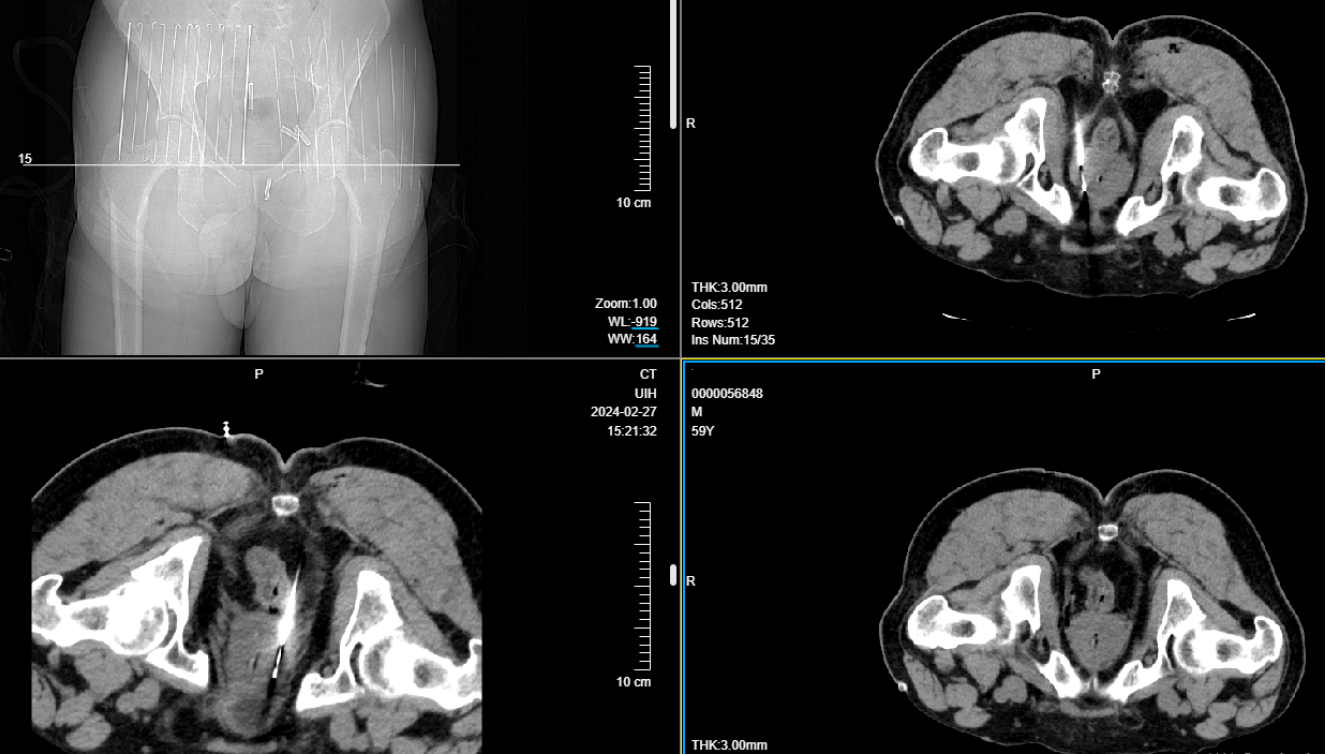

4.
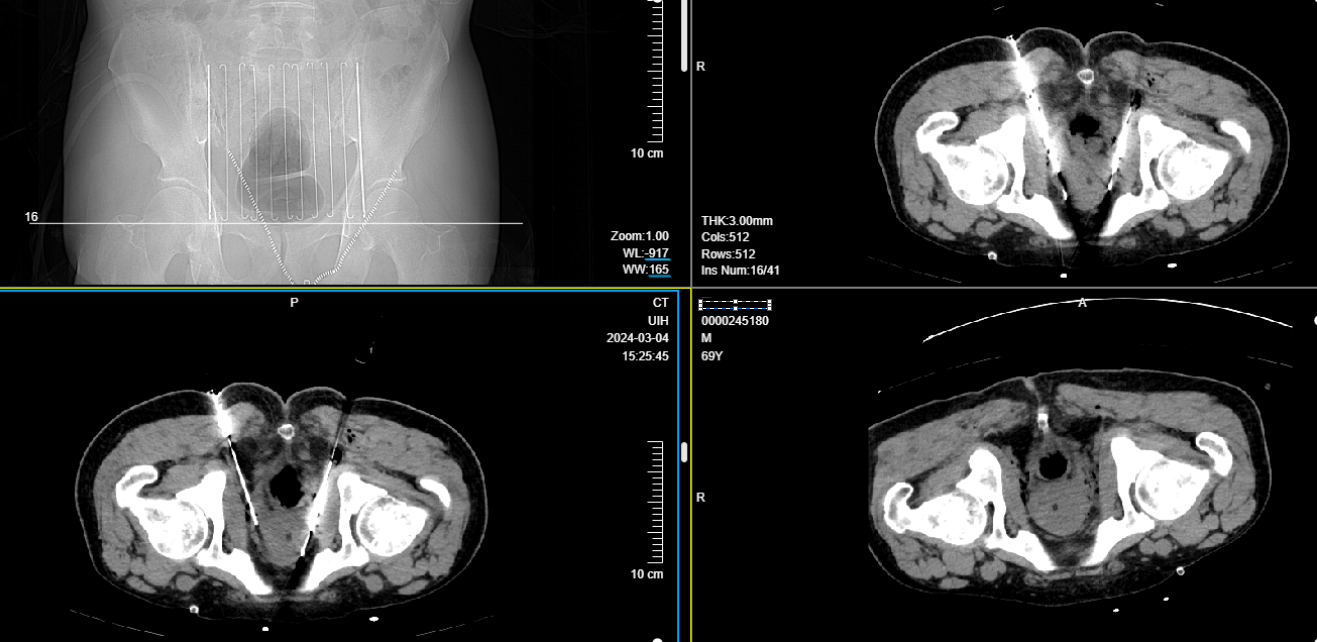

5.
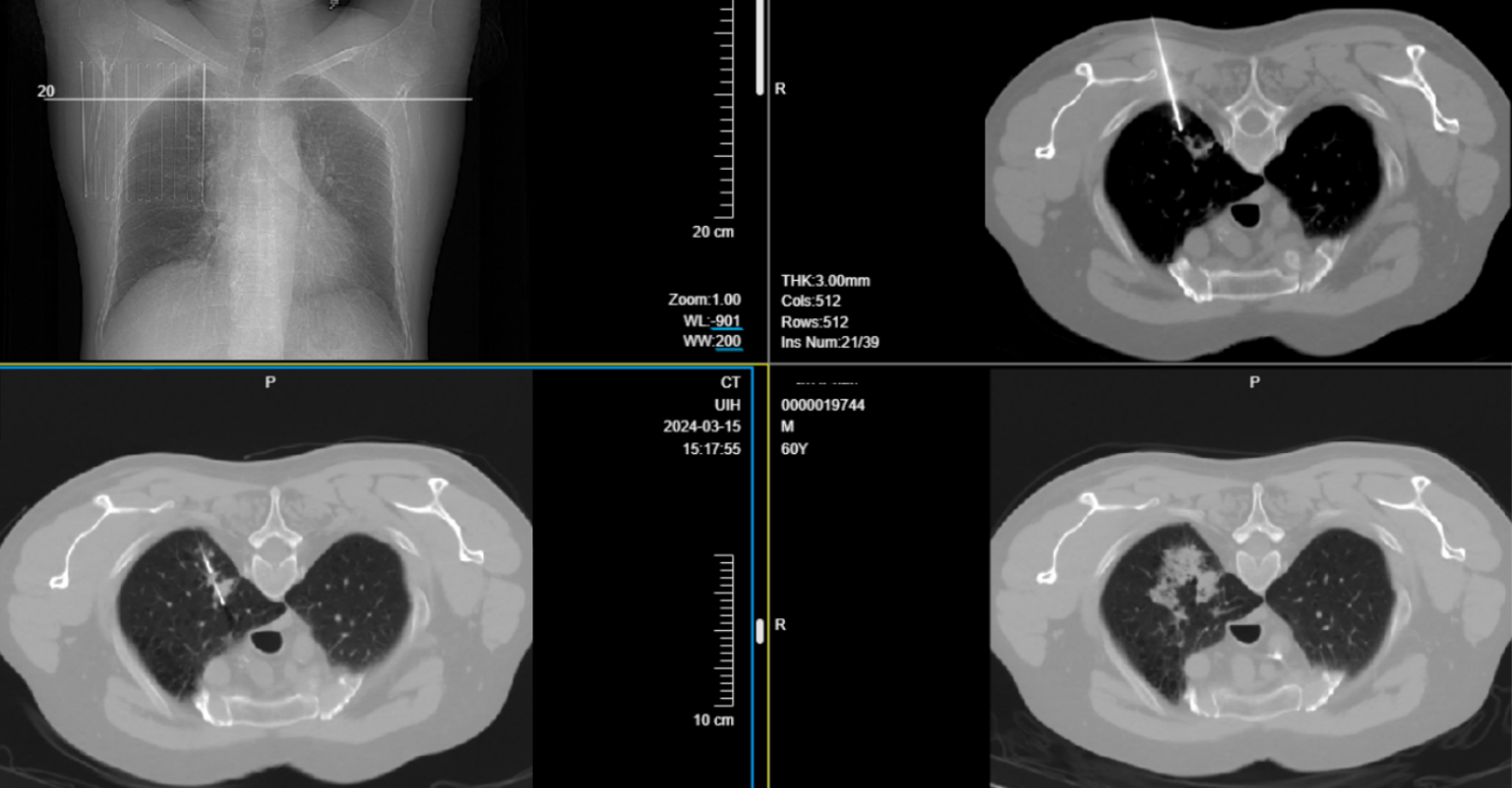

6.
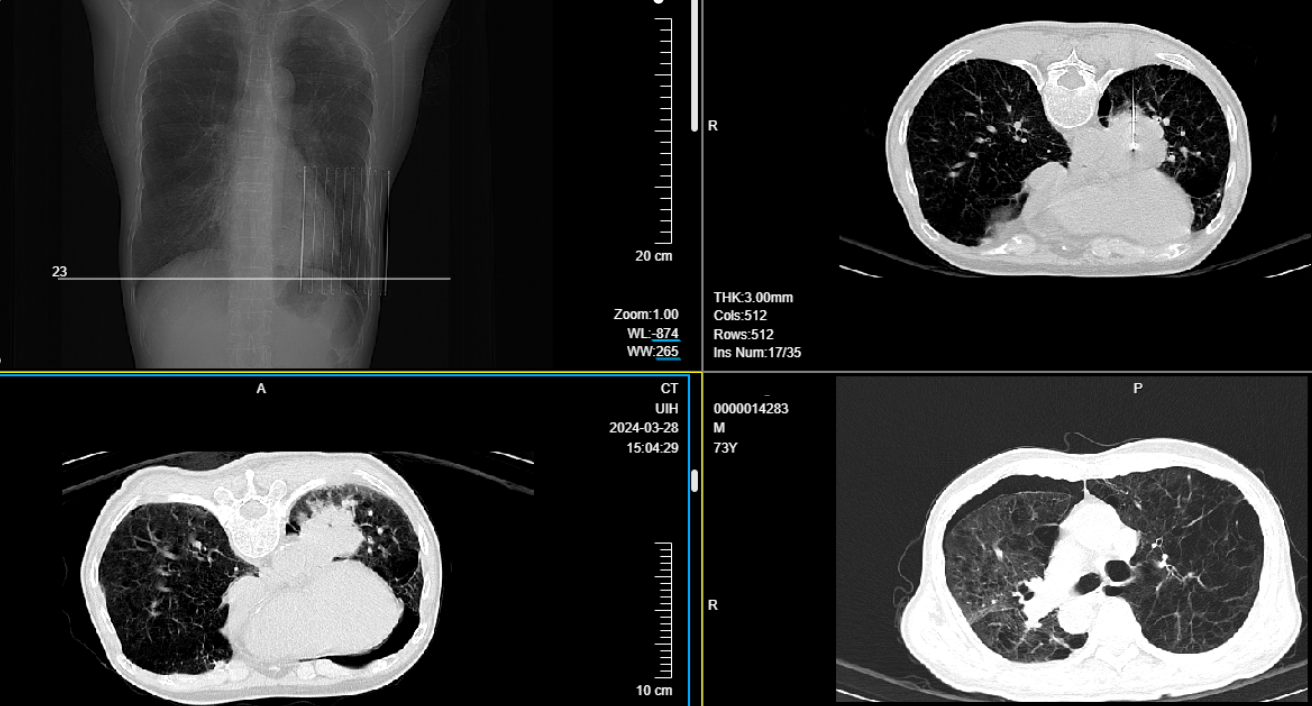

7.
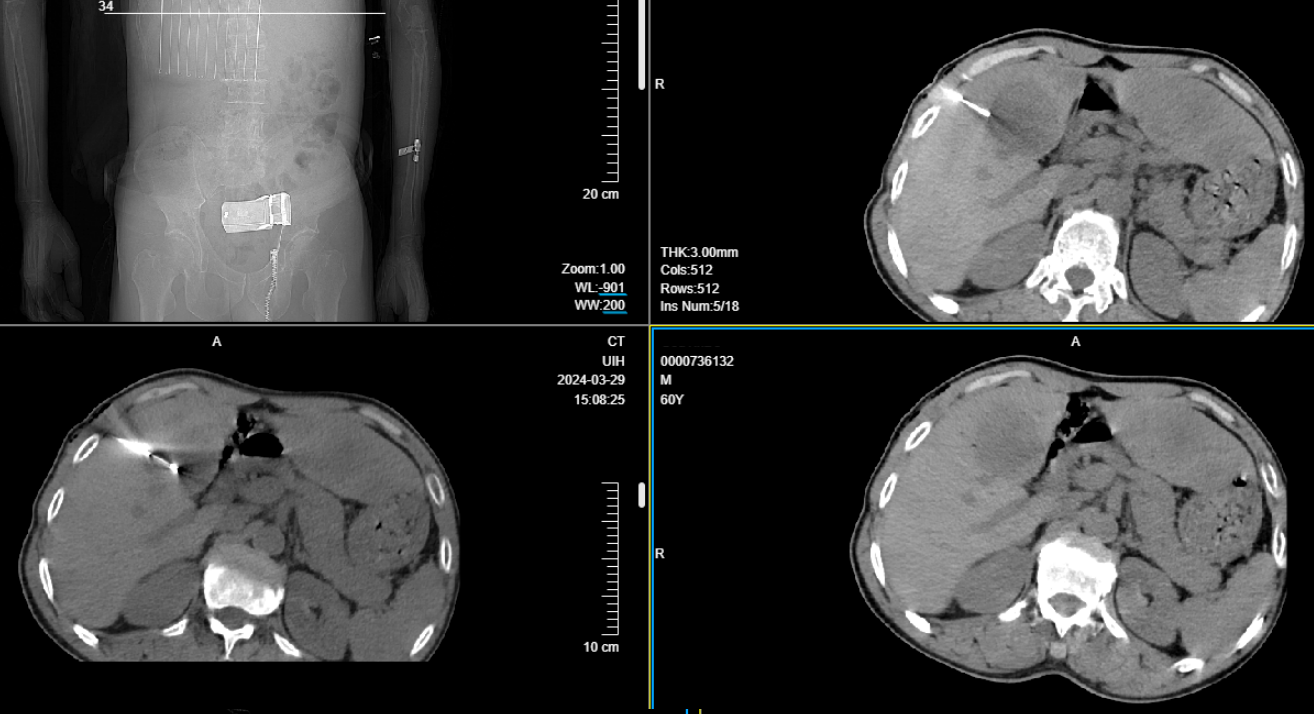

8.
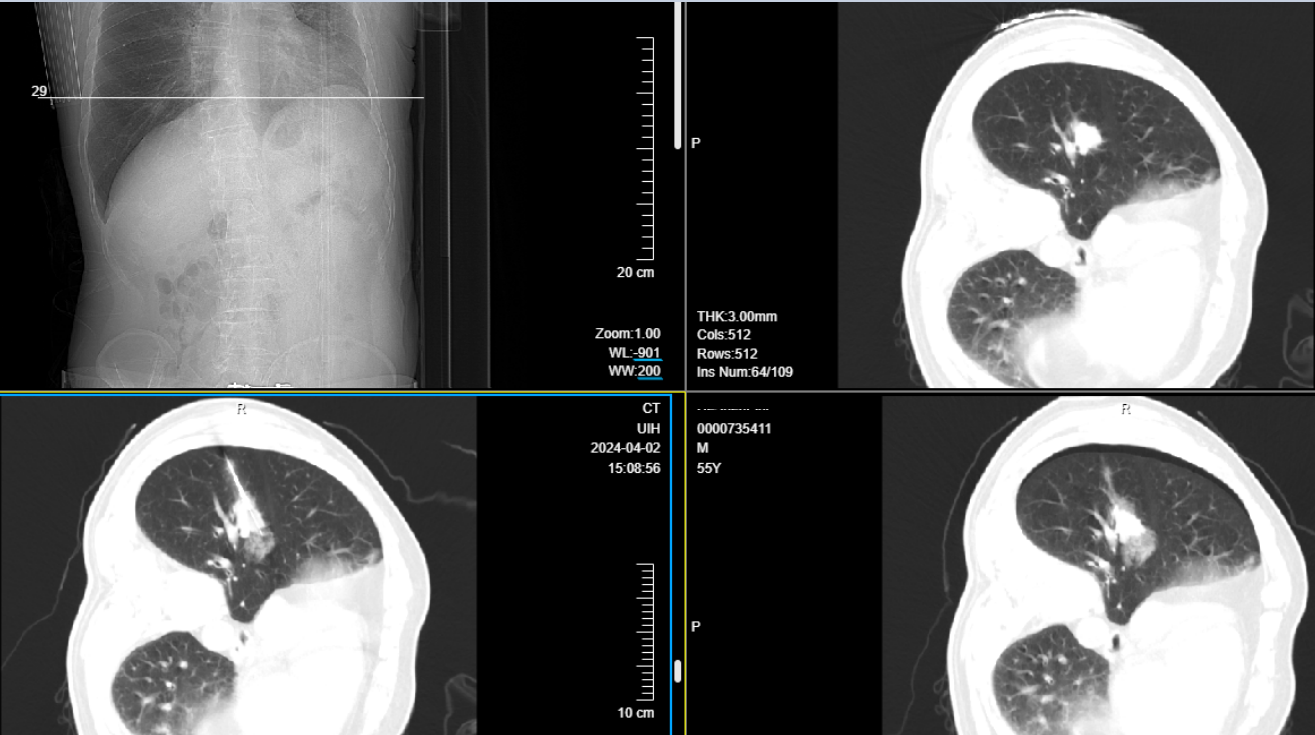

9.
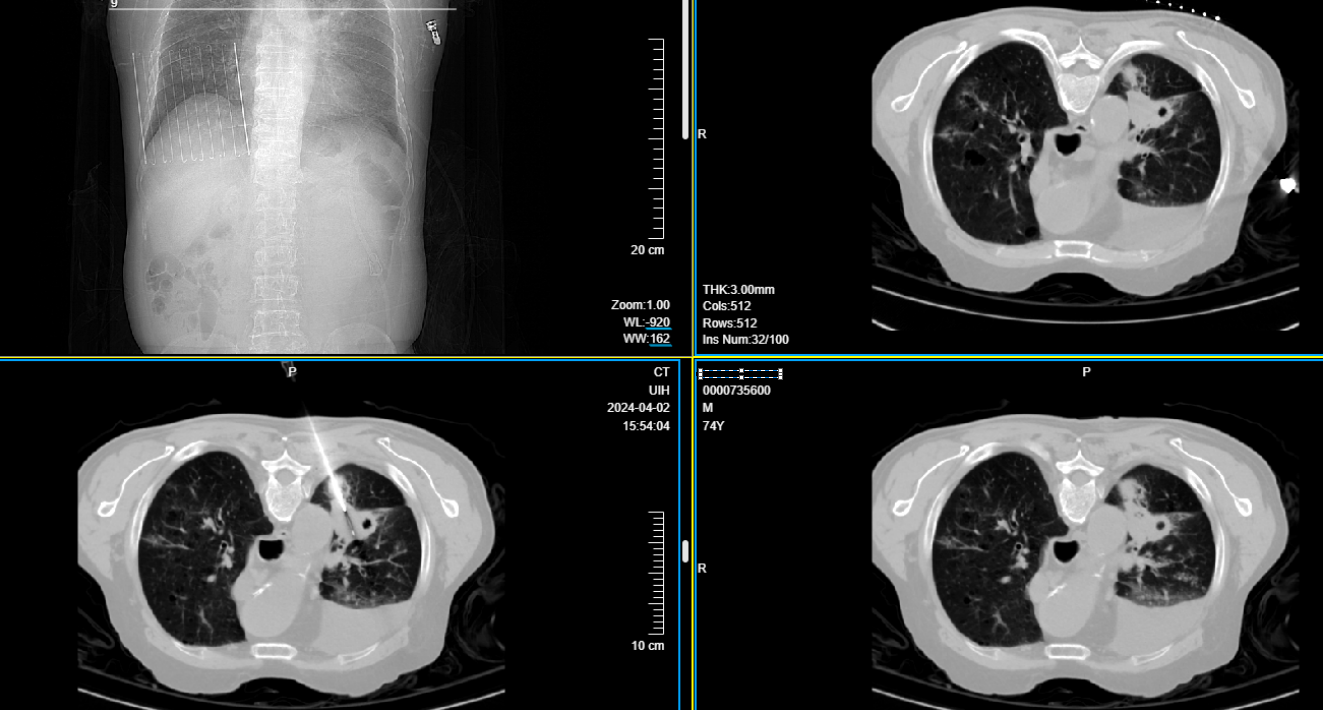

10.
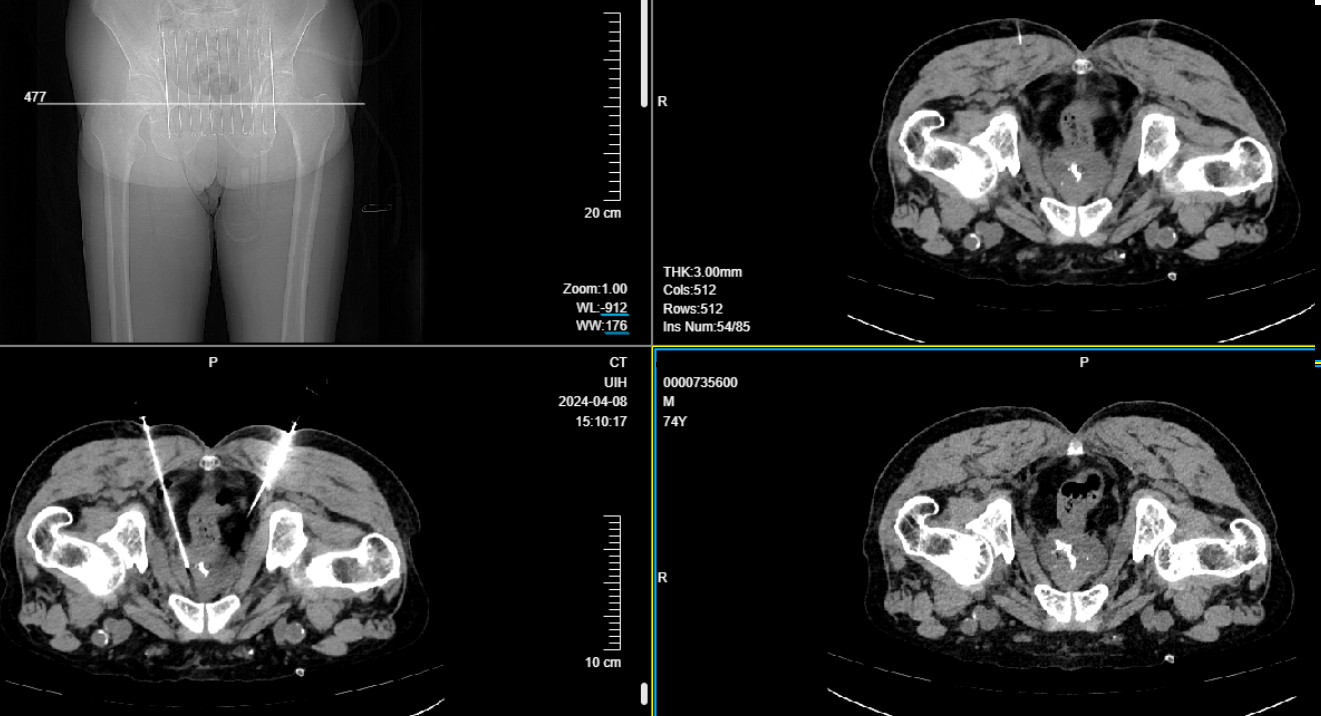

11.
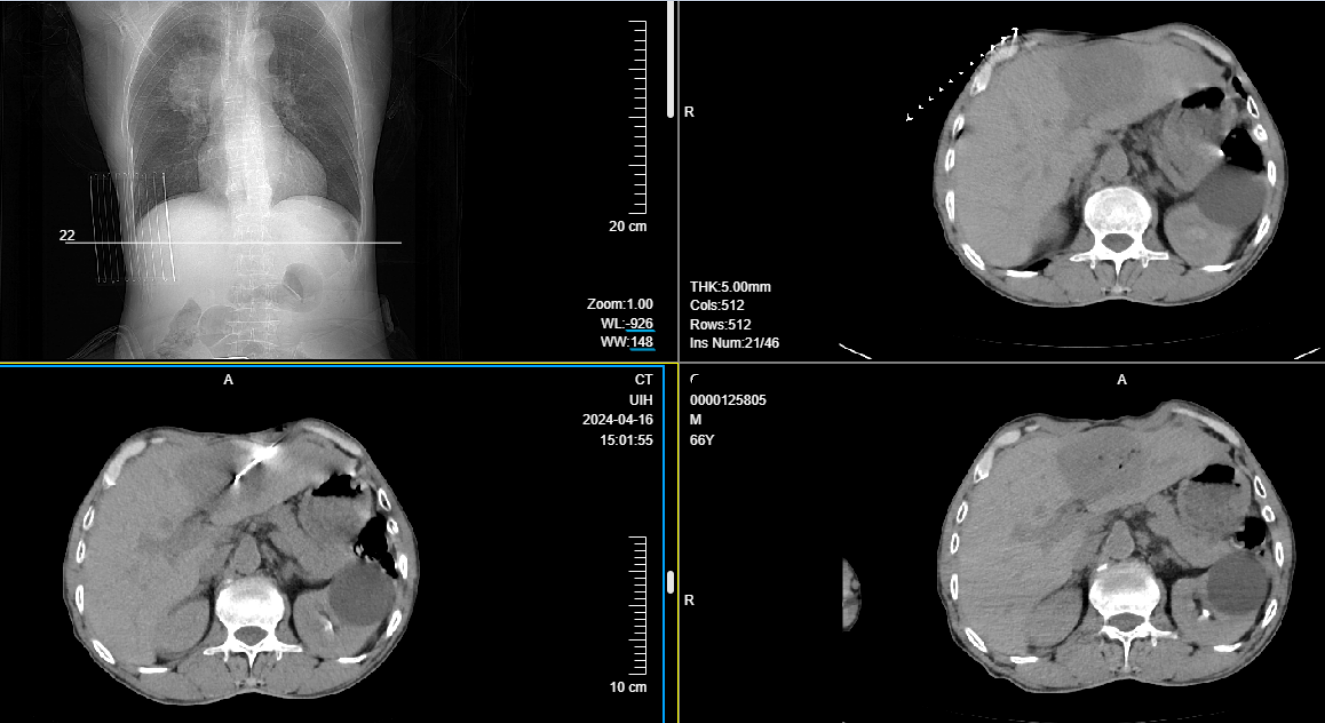

12.
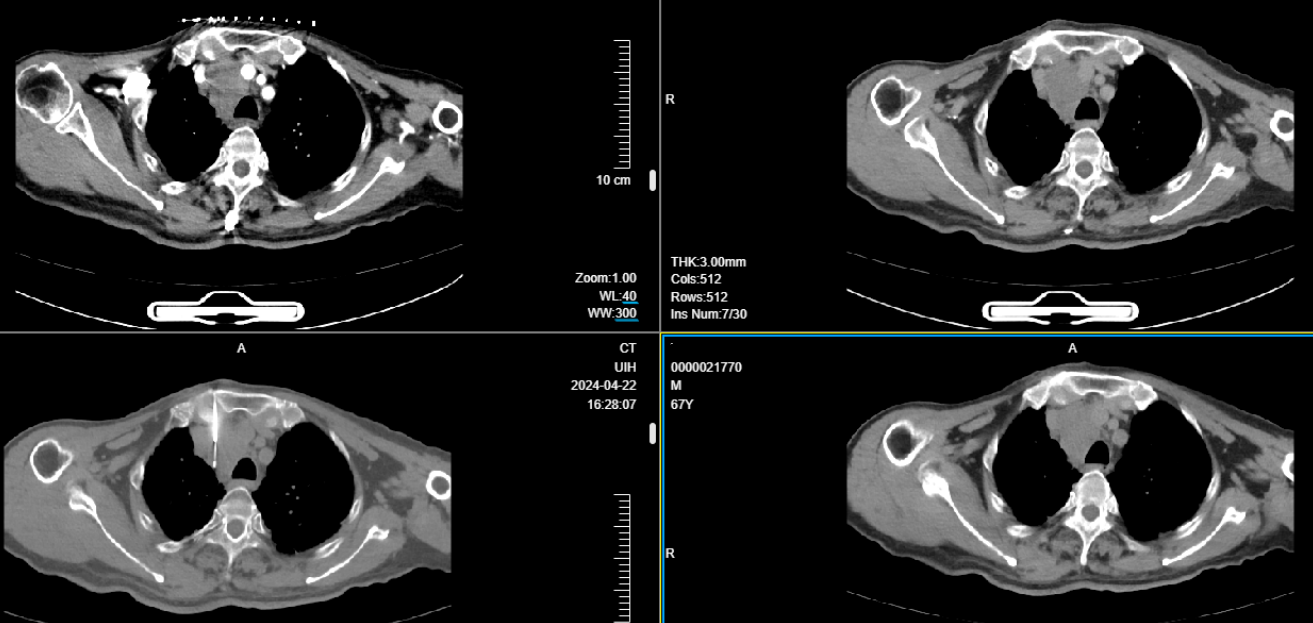

13.
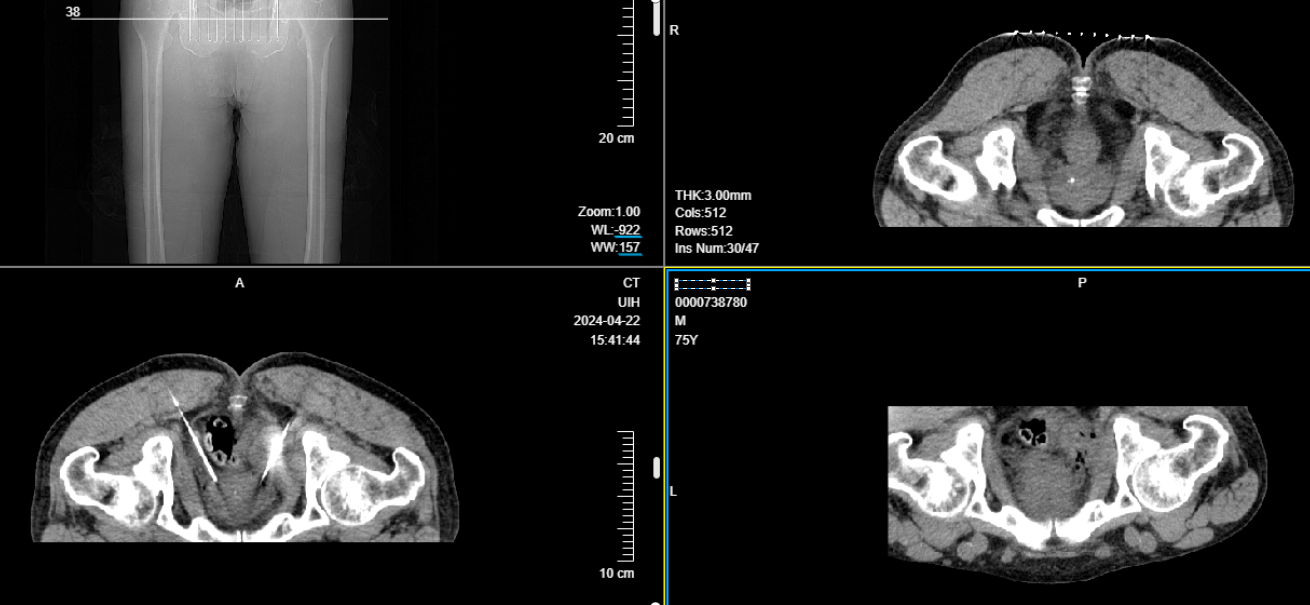

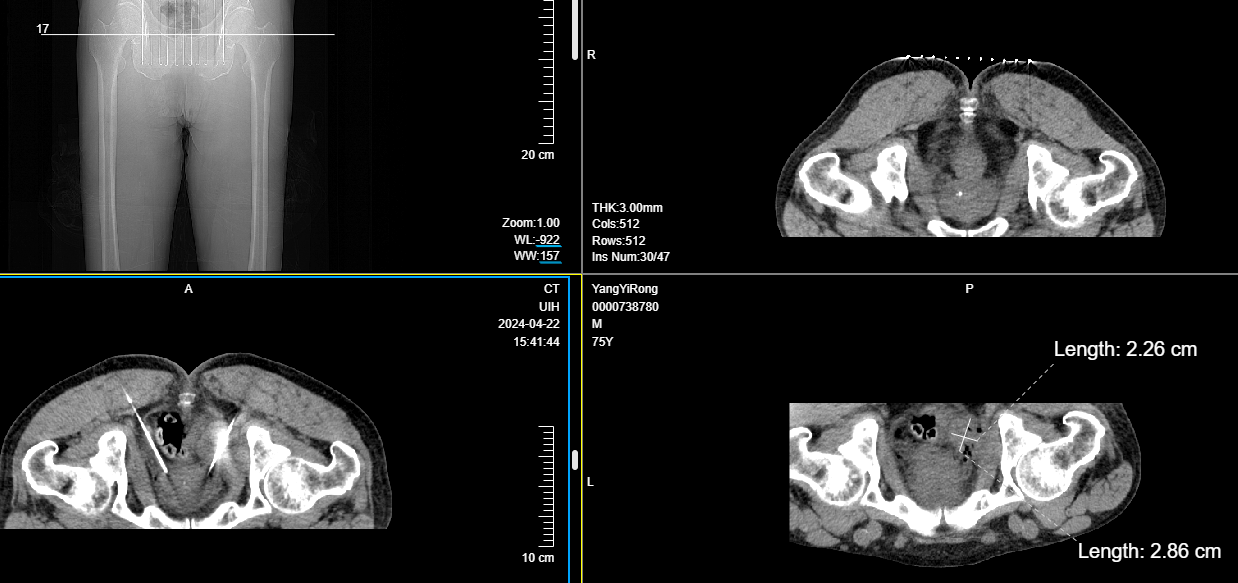

14.
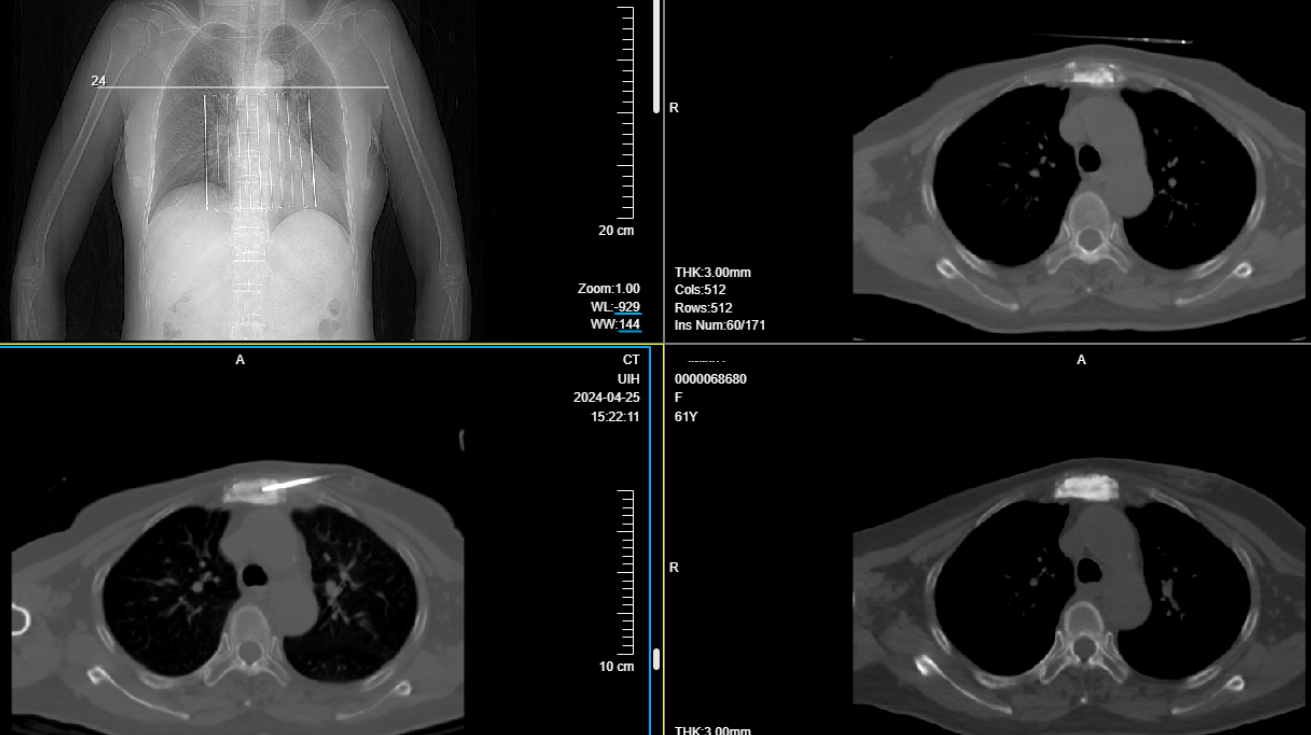

15.
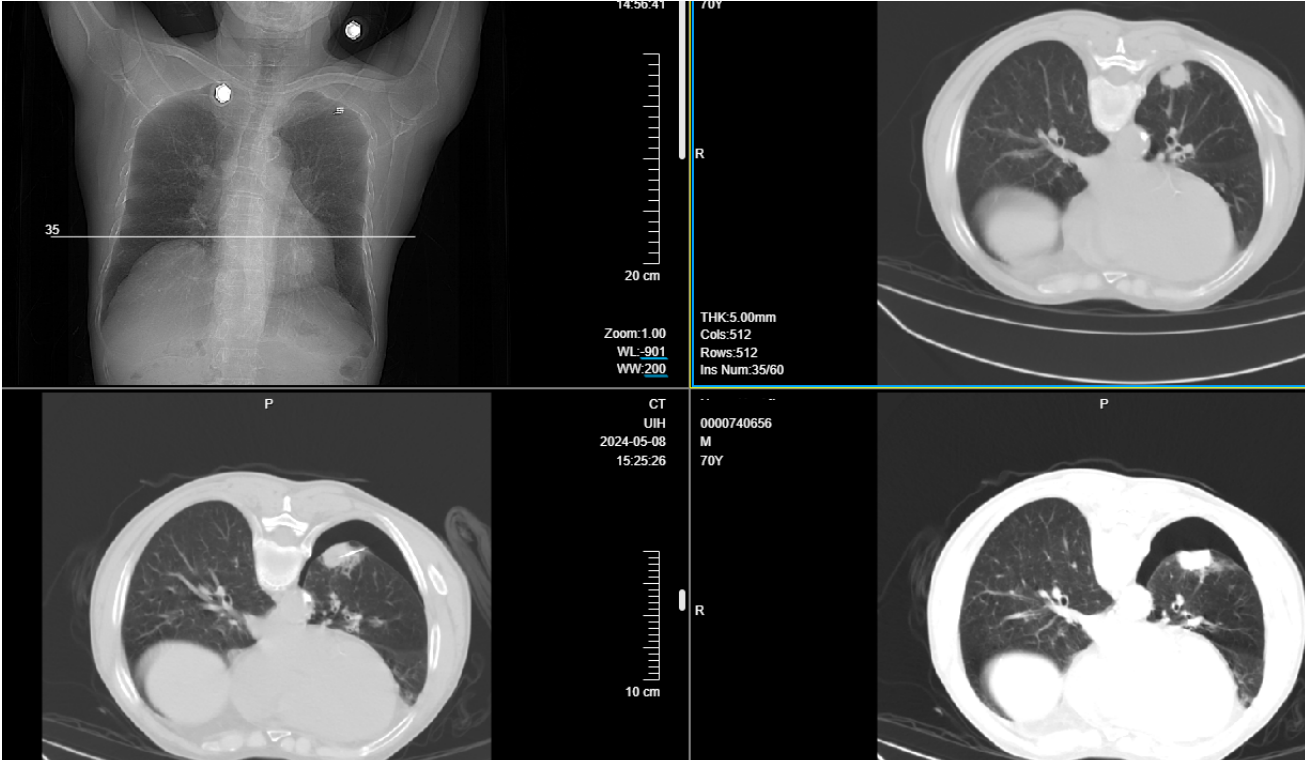

16.
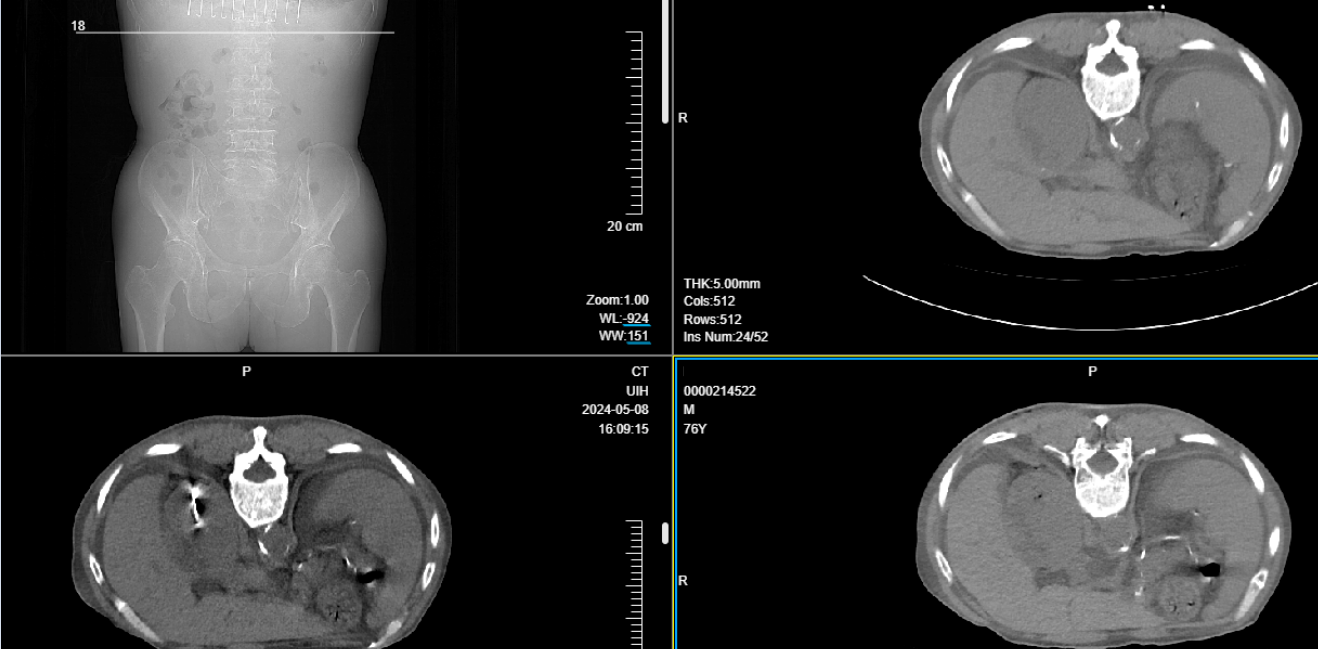

17.
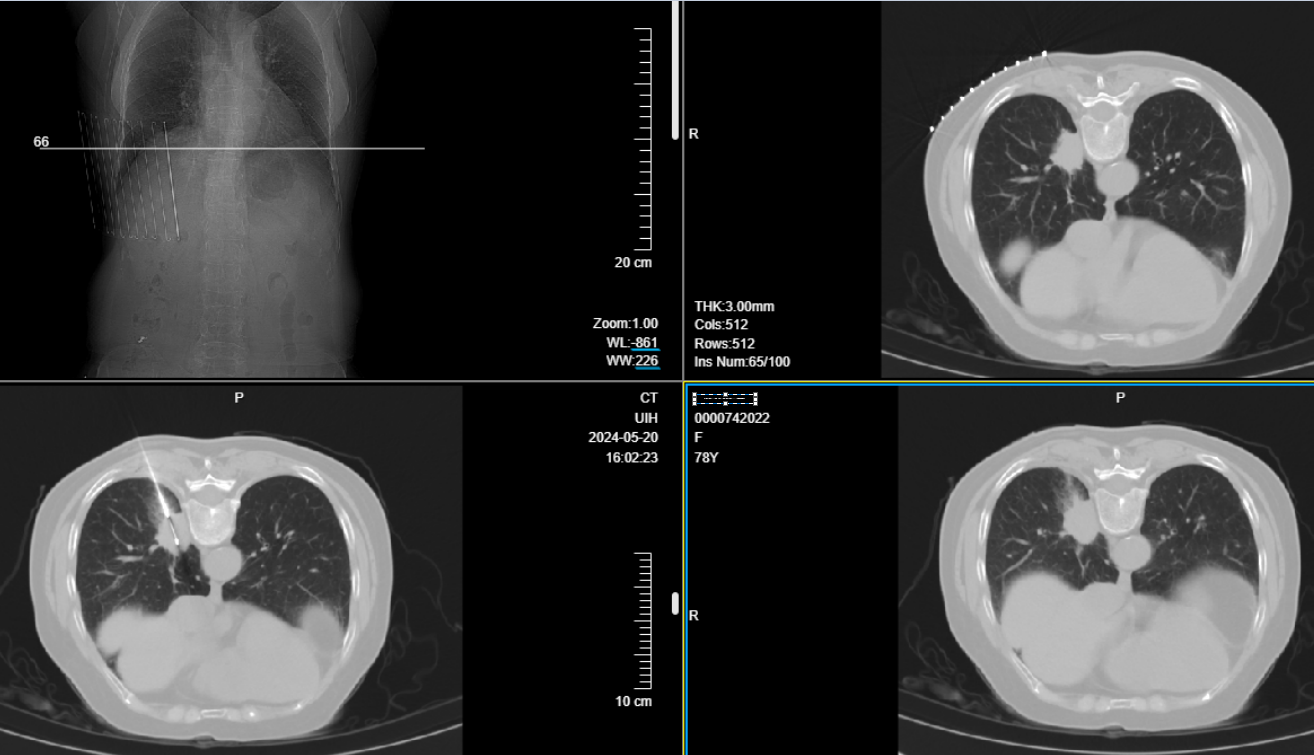

18.
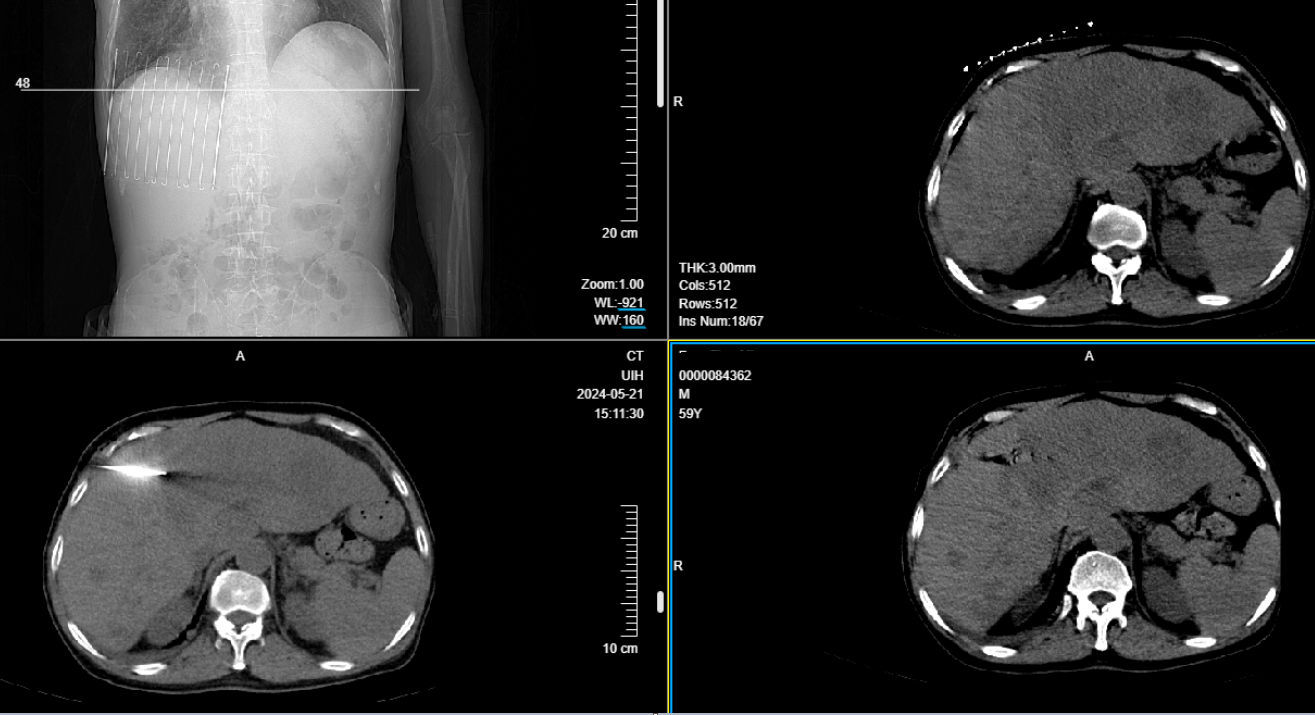

19.
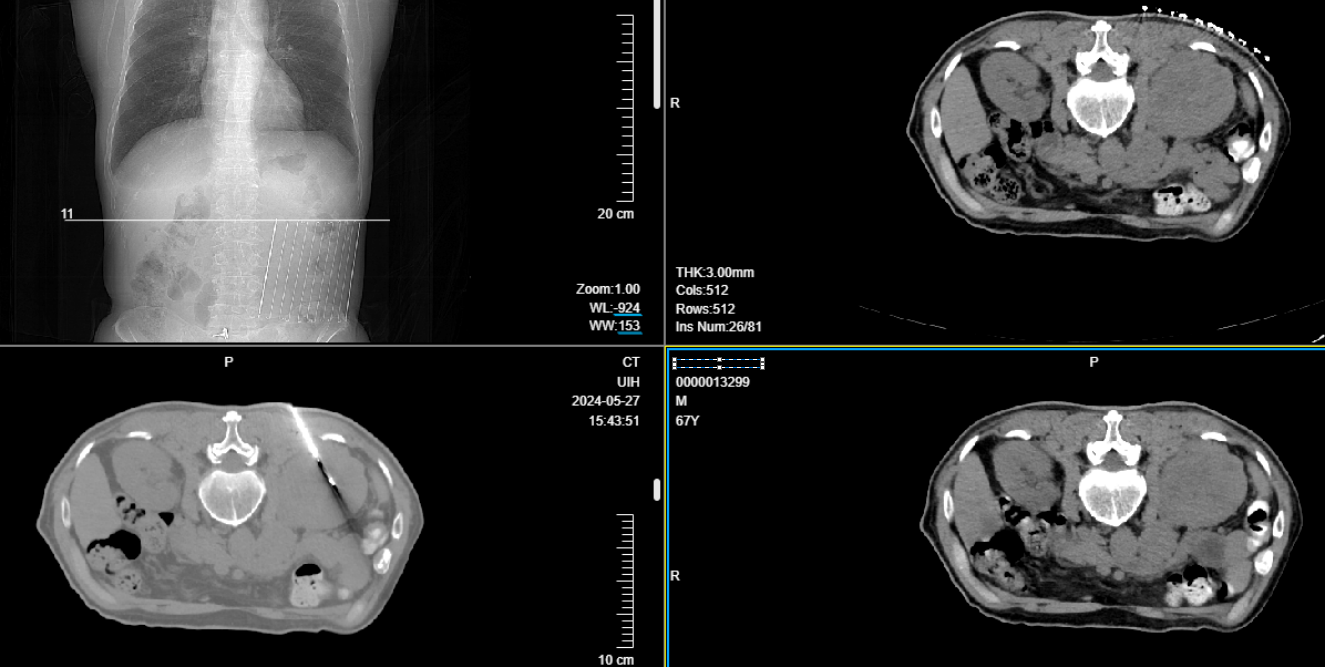

20.
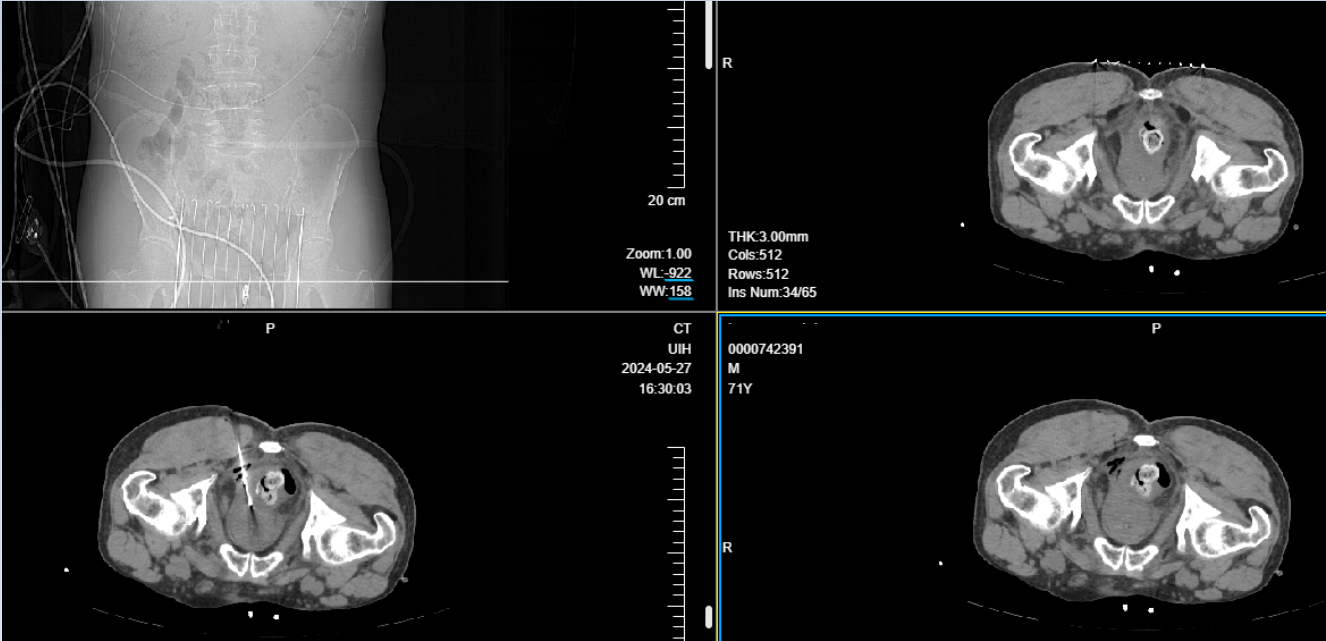

21.
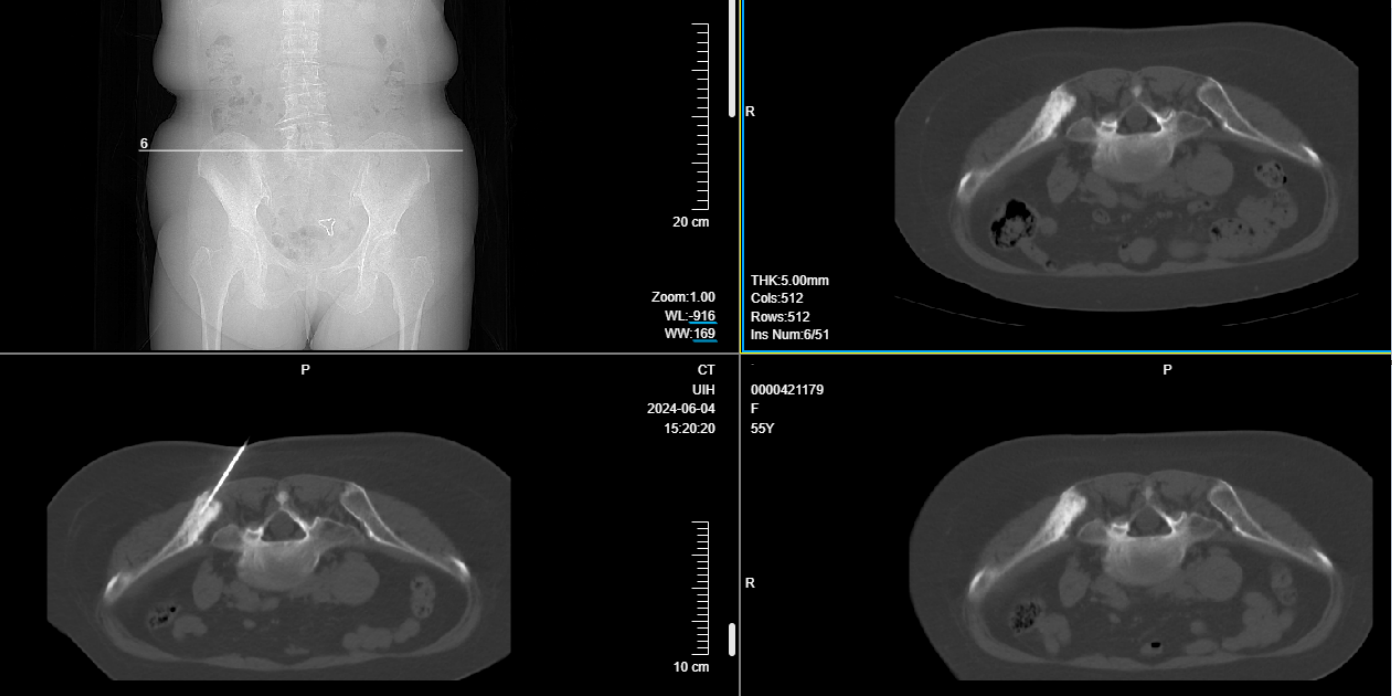

22.
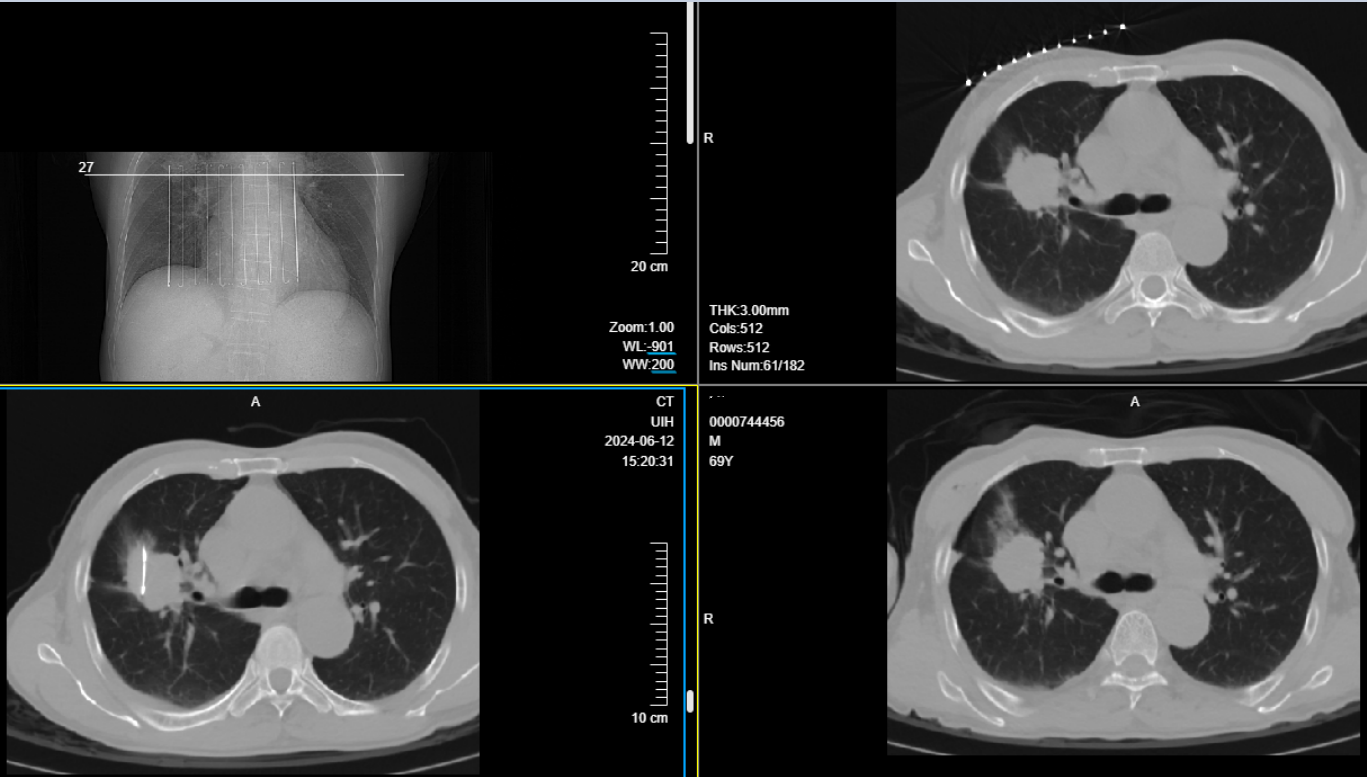

23.
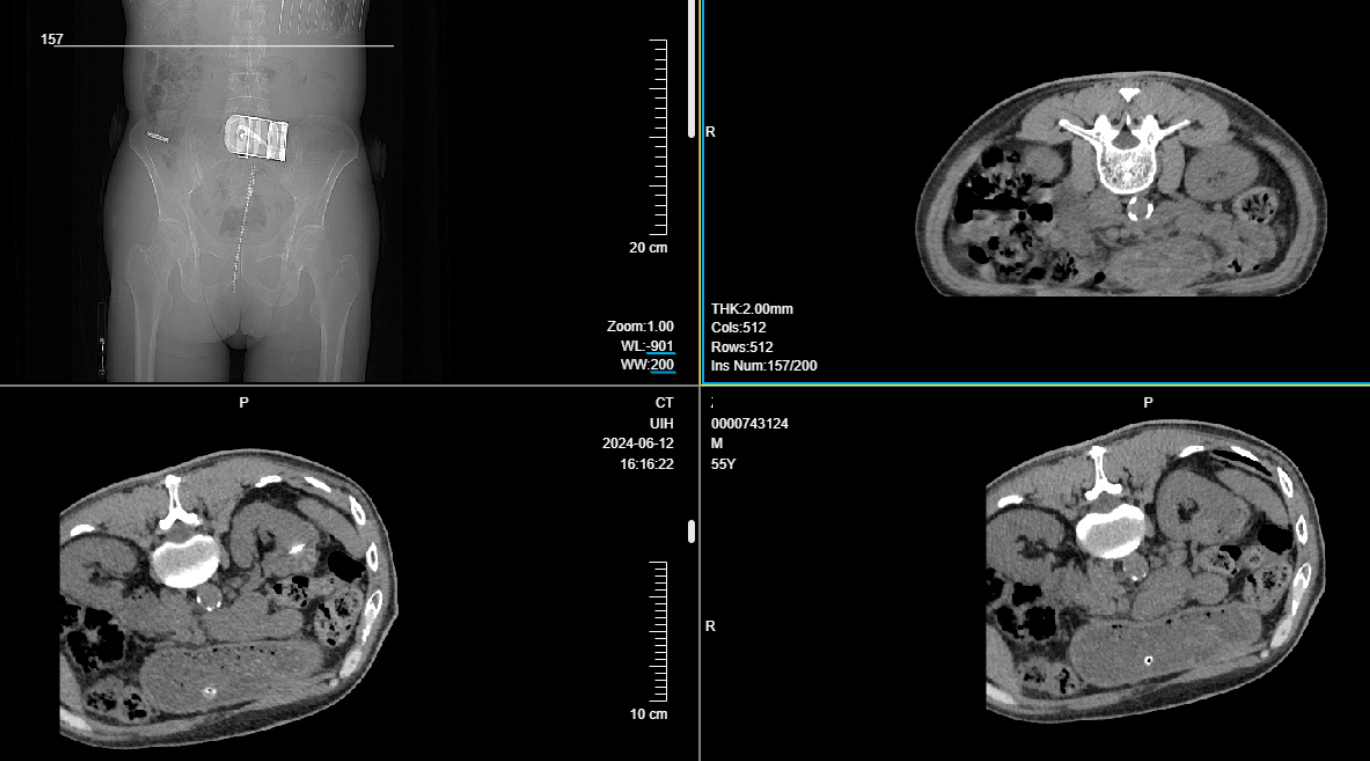

24.
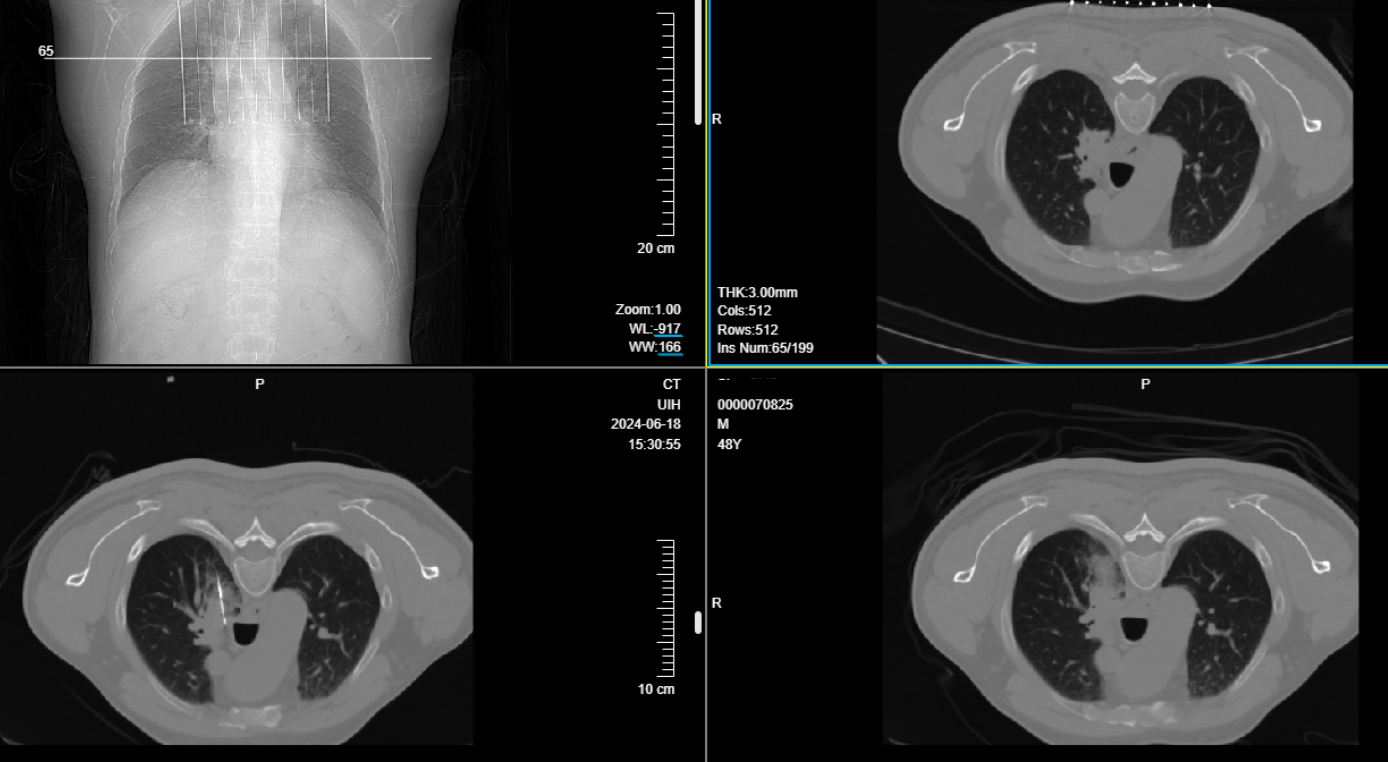

25.
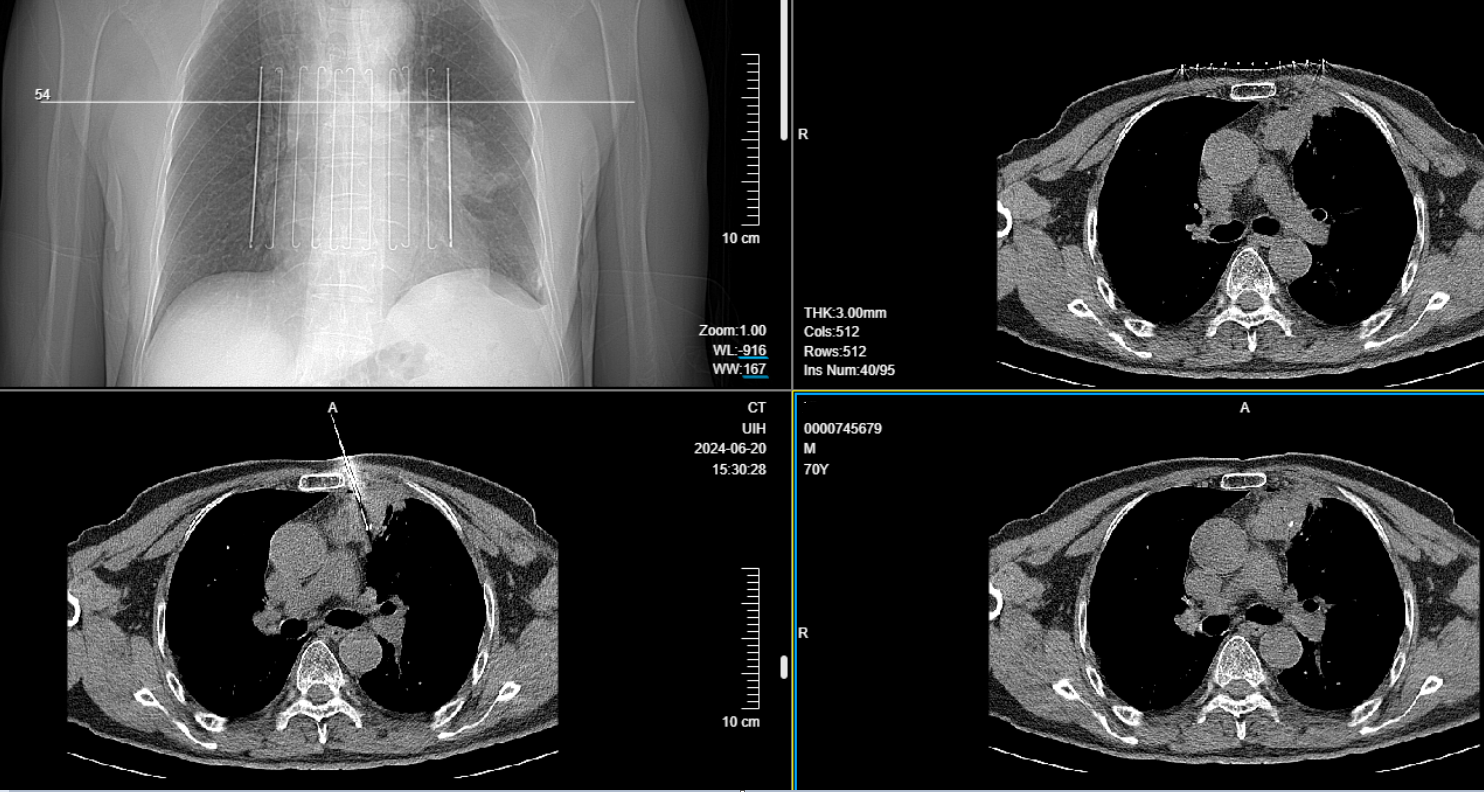

26.
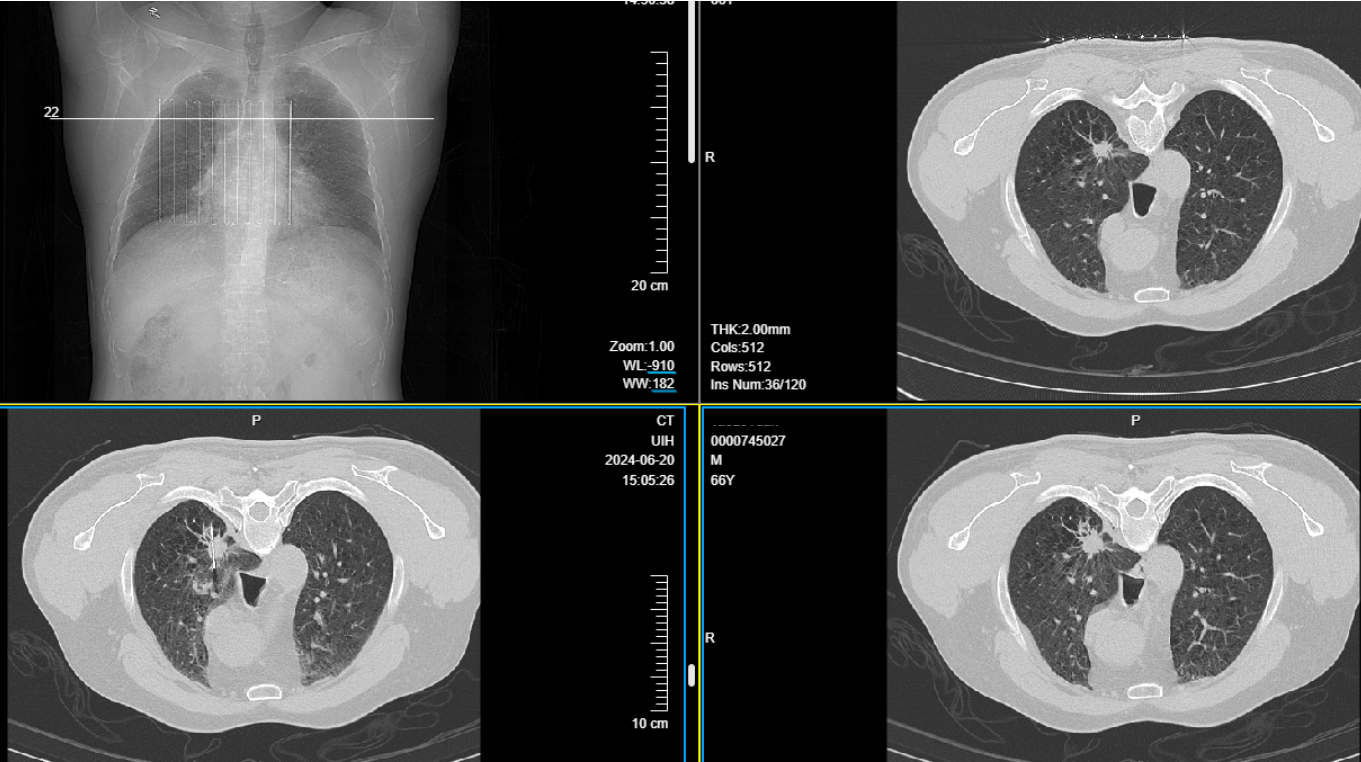

27.
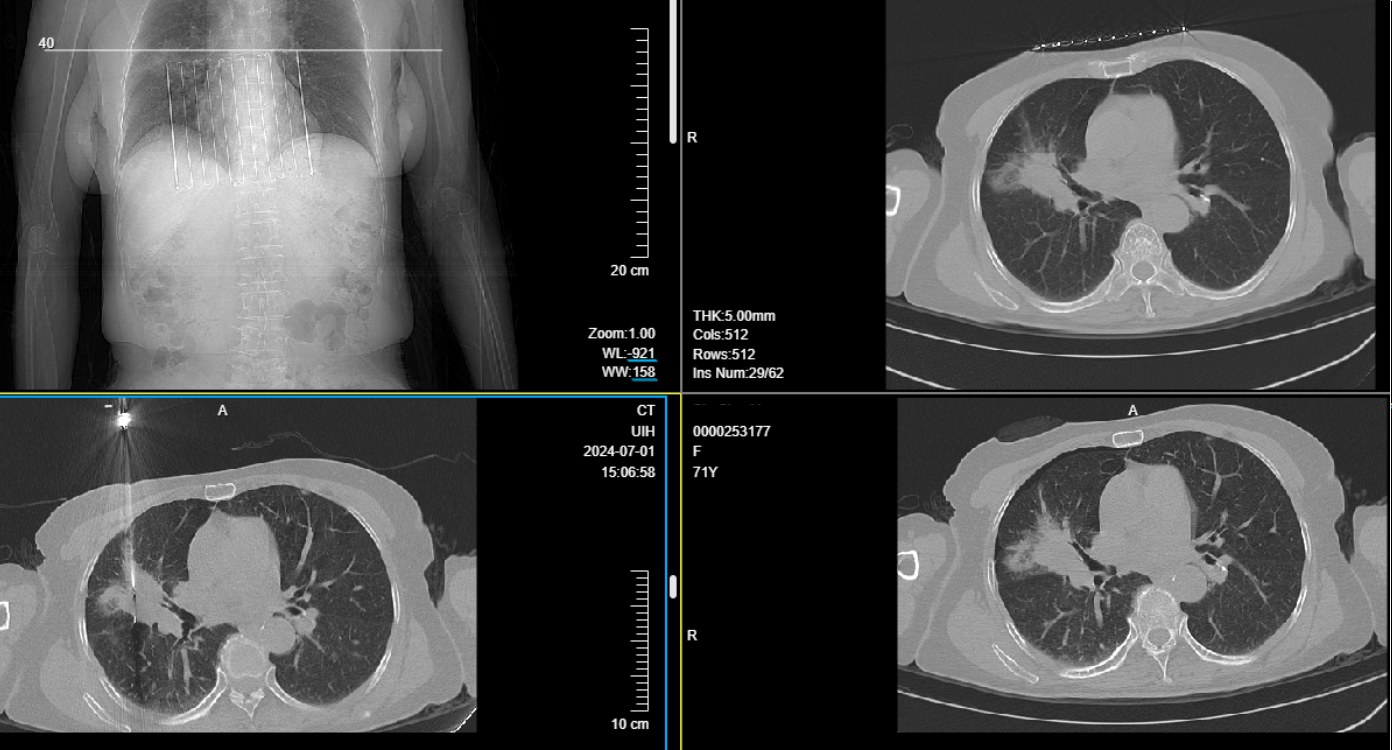

28.
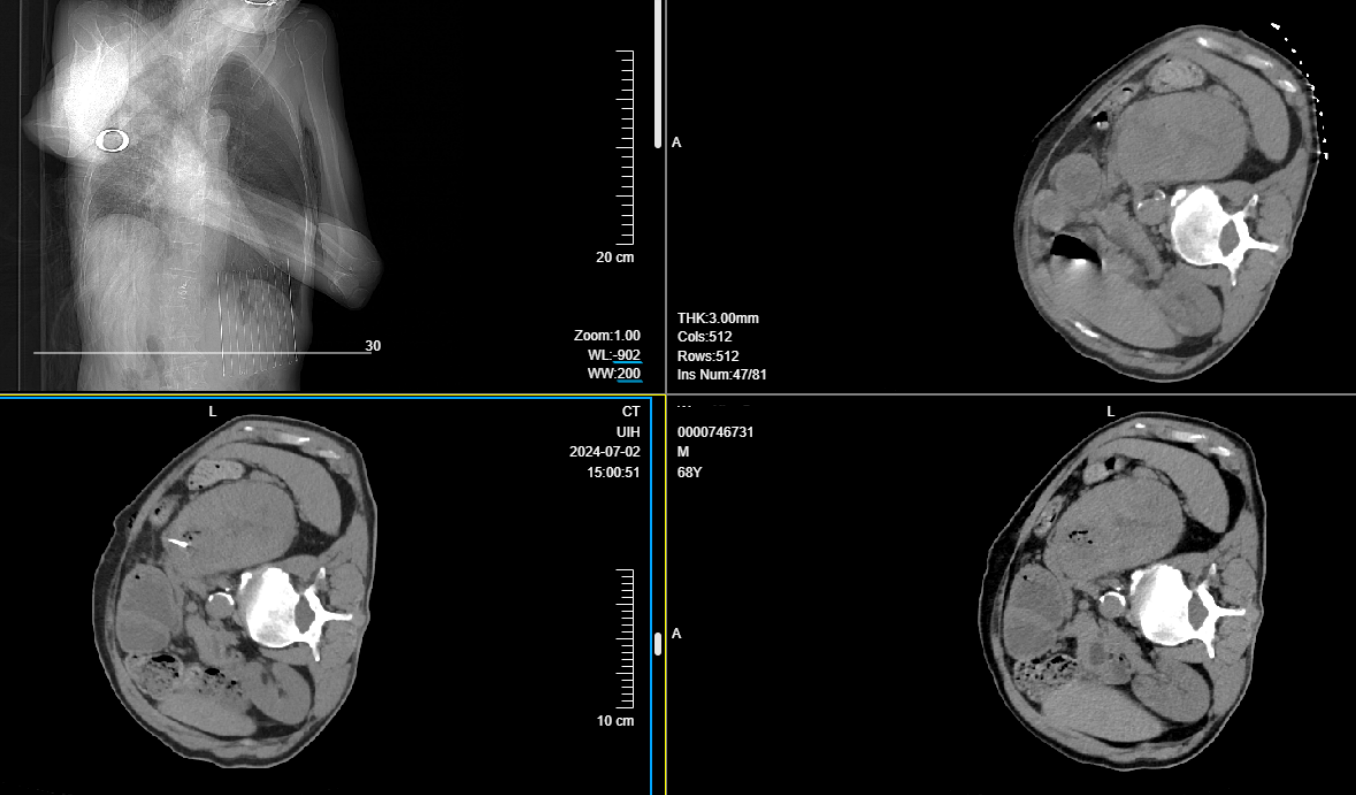

29.
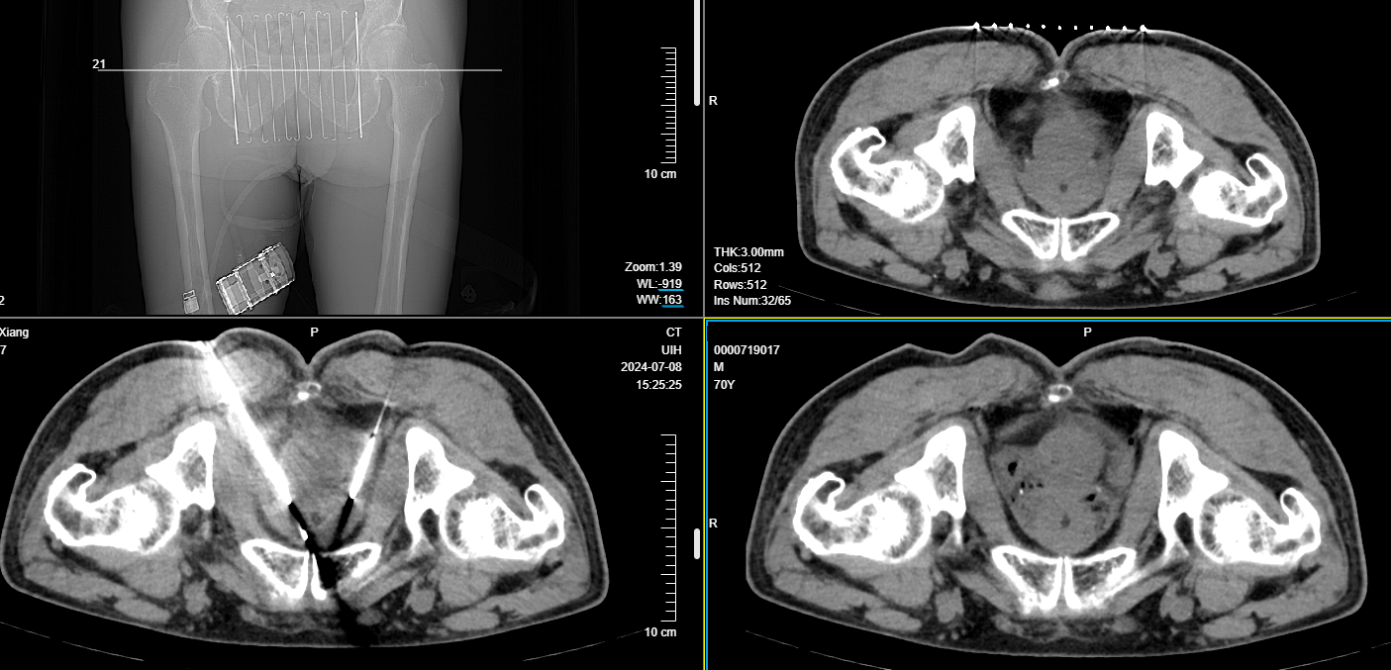

30.
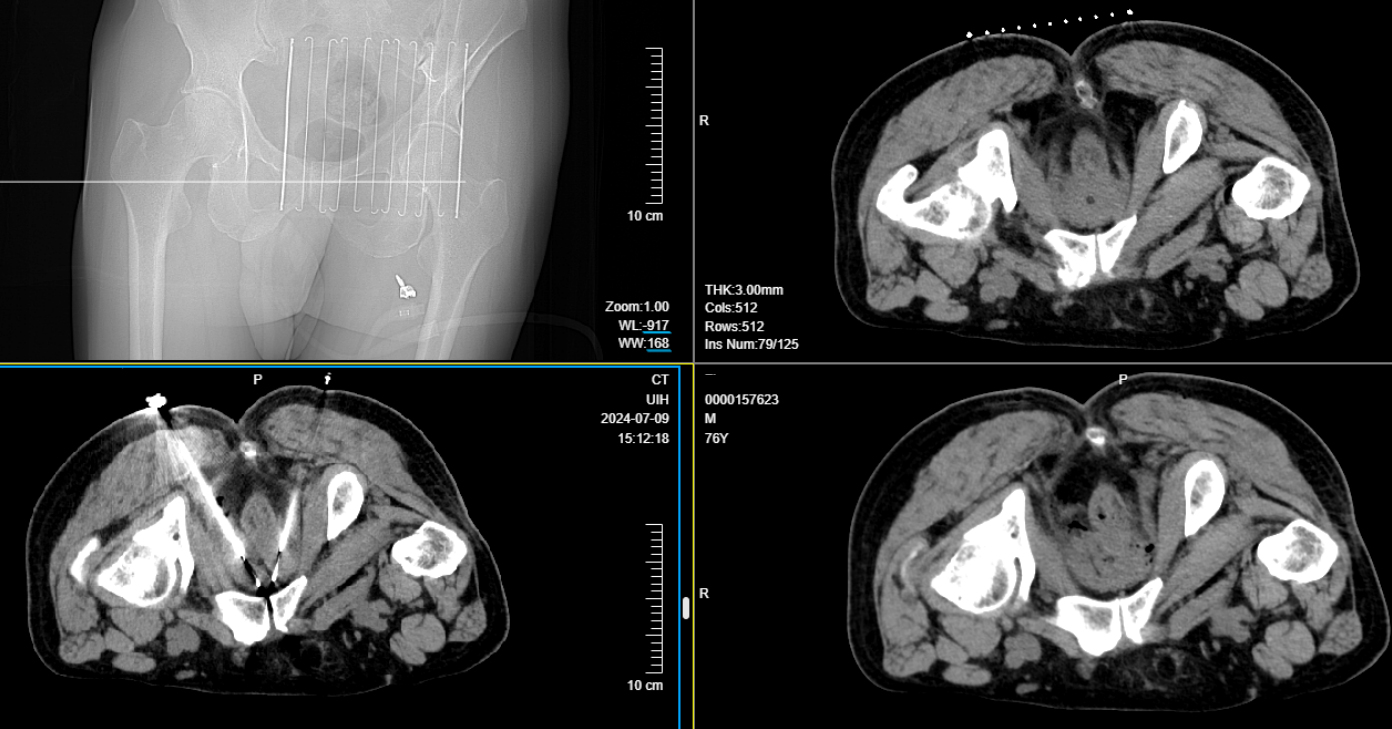

31.
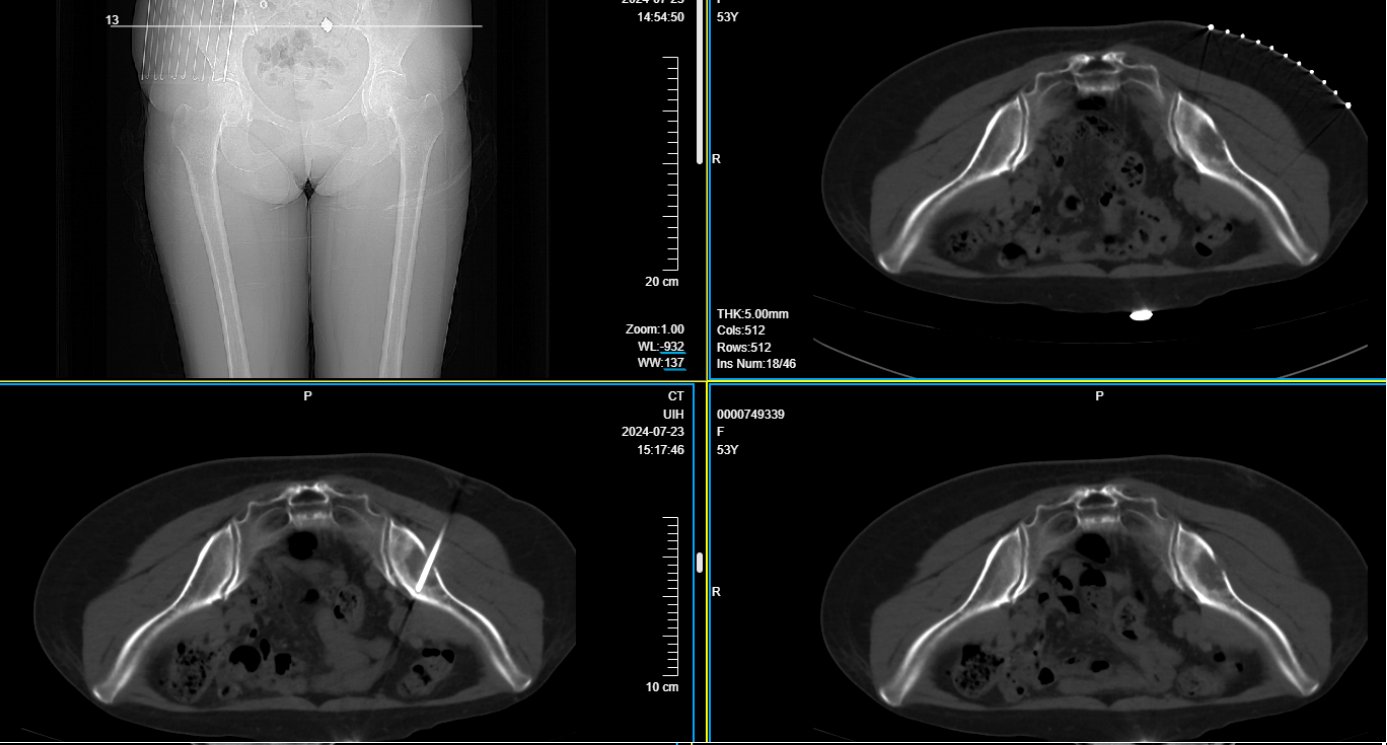

32.
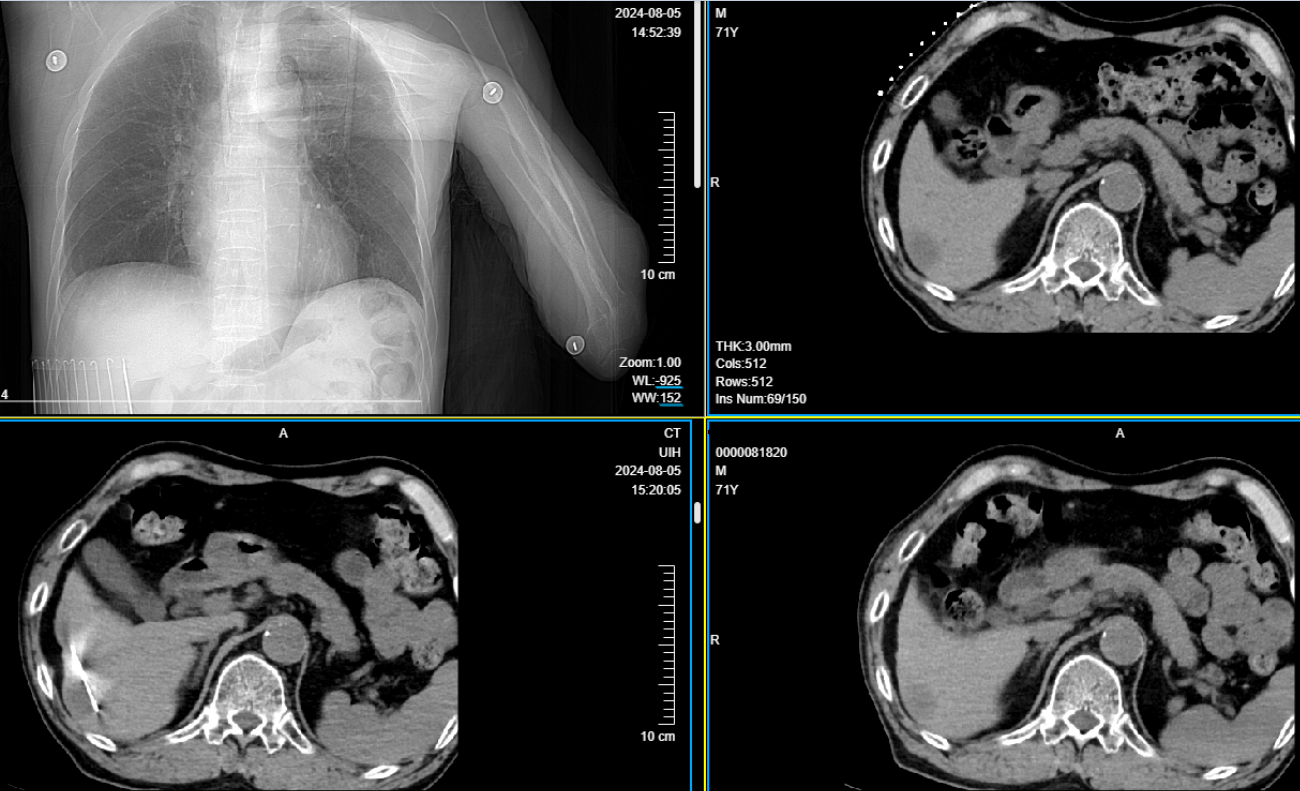

33.
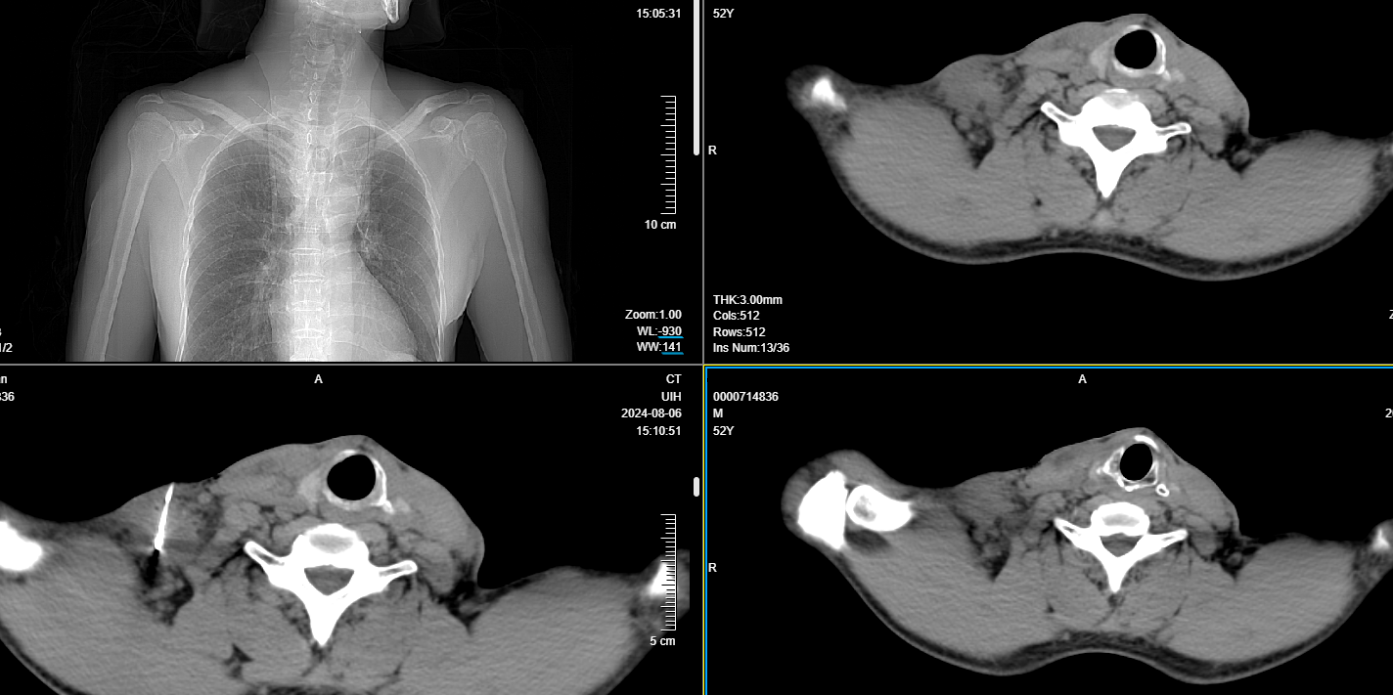

34.
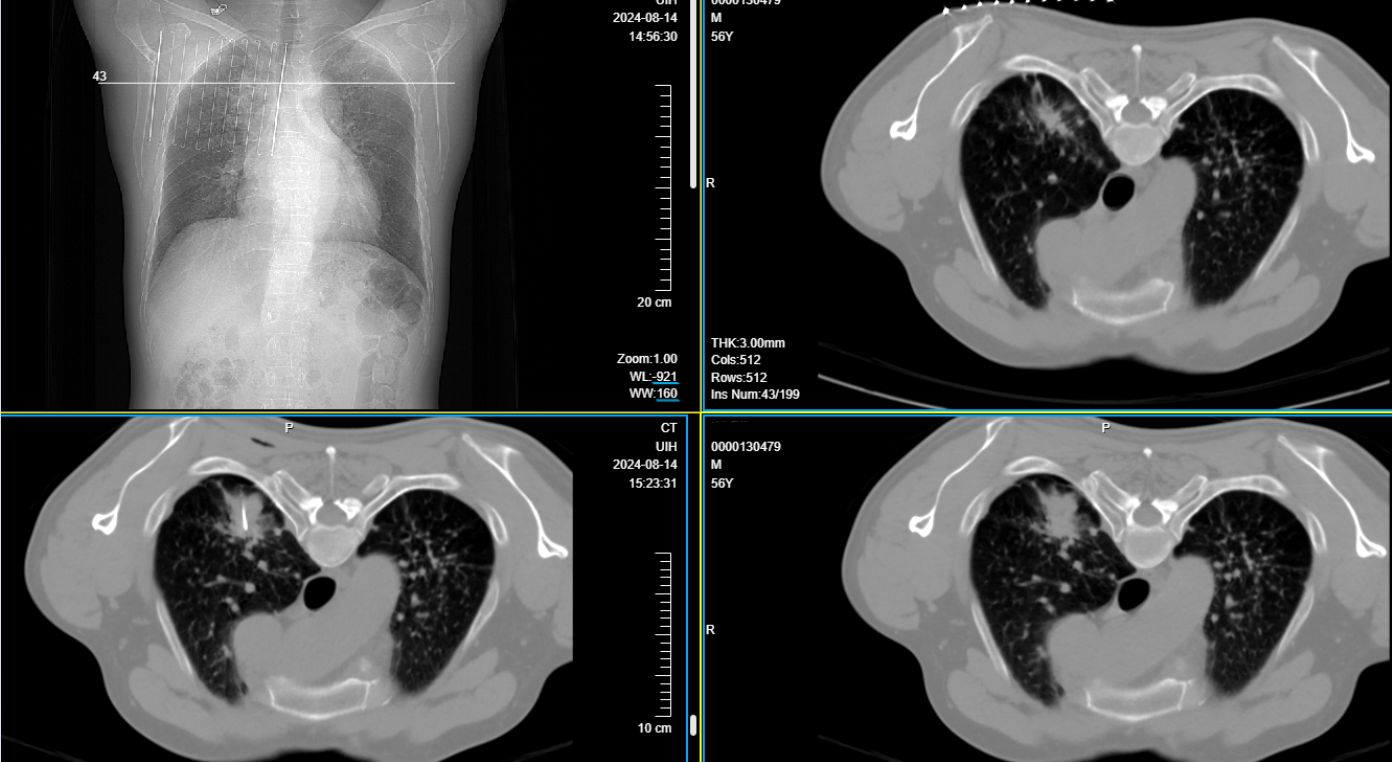

35.
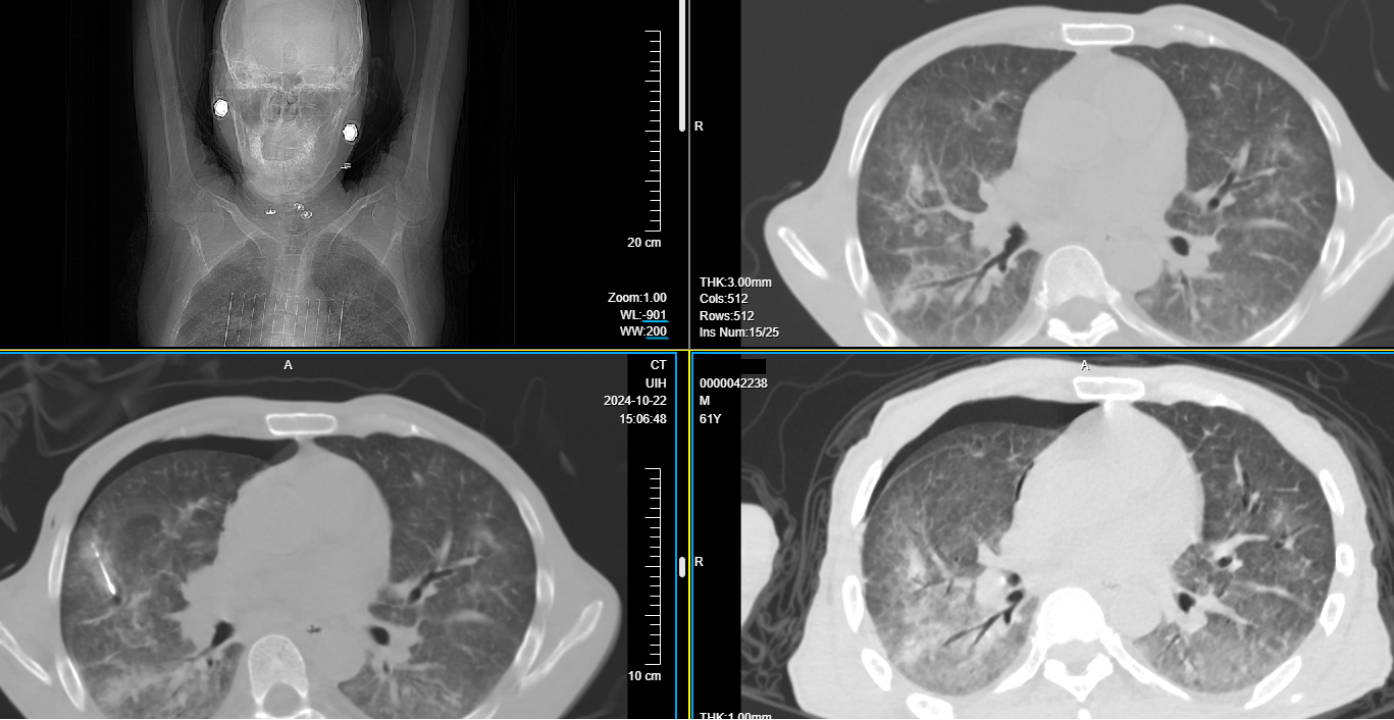

36.
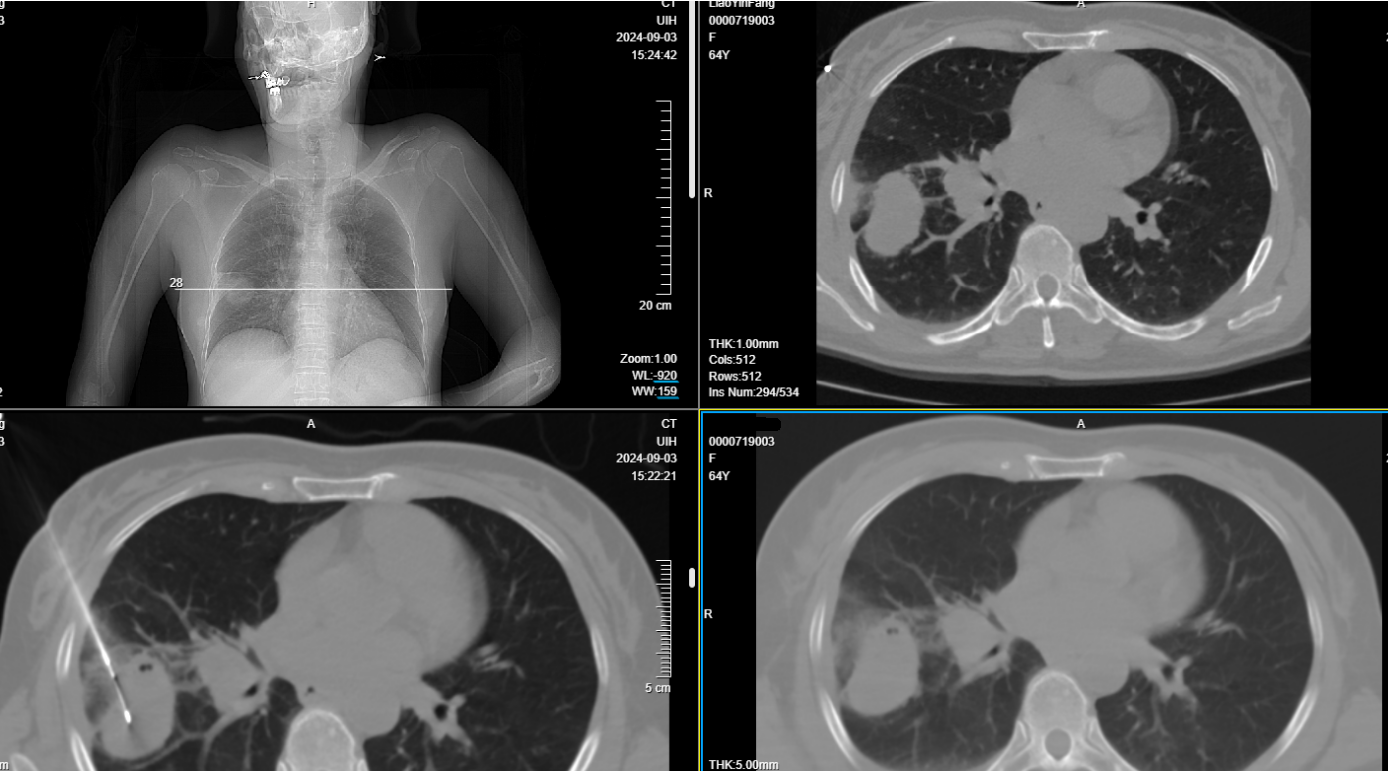

37.
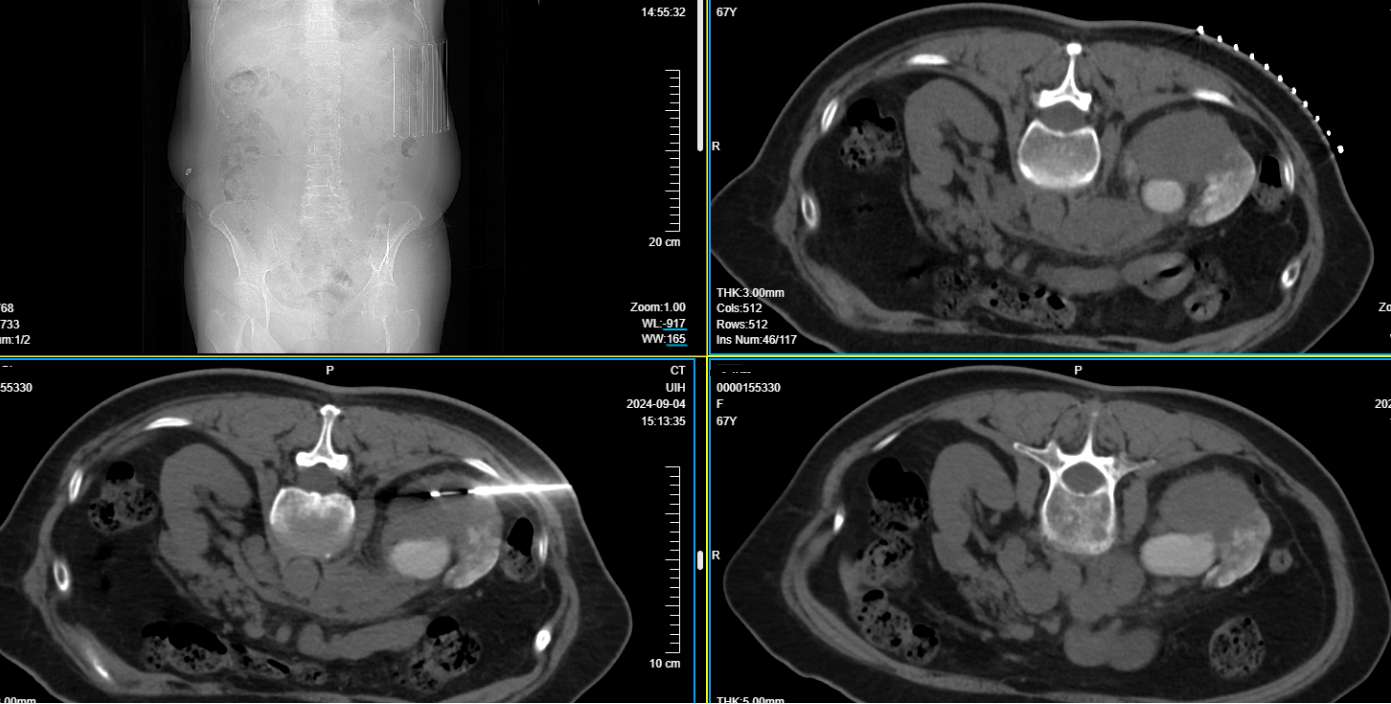

38.
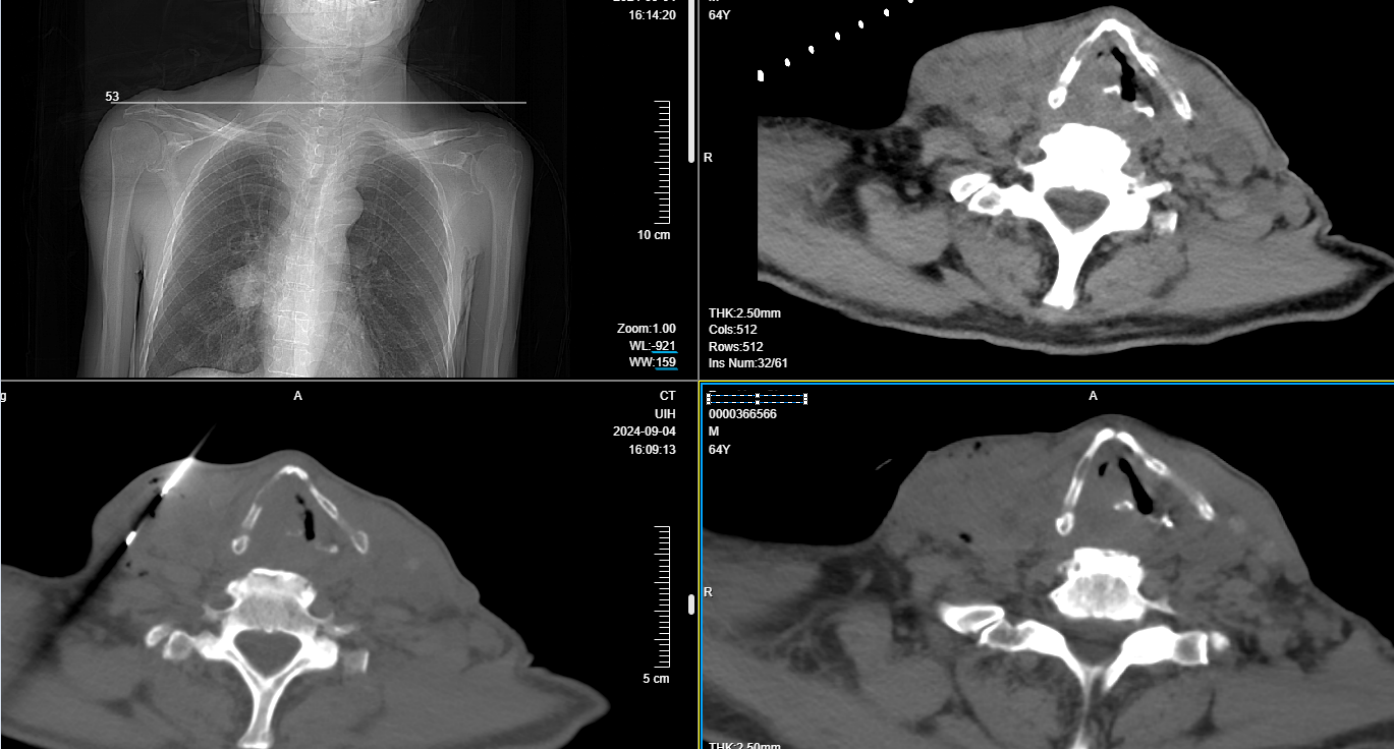

39.
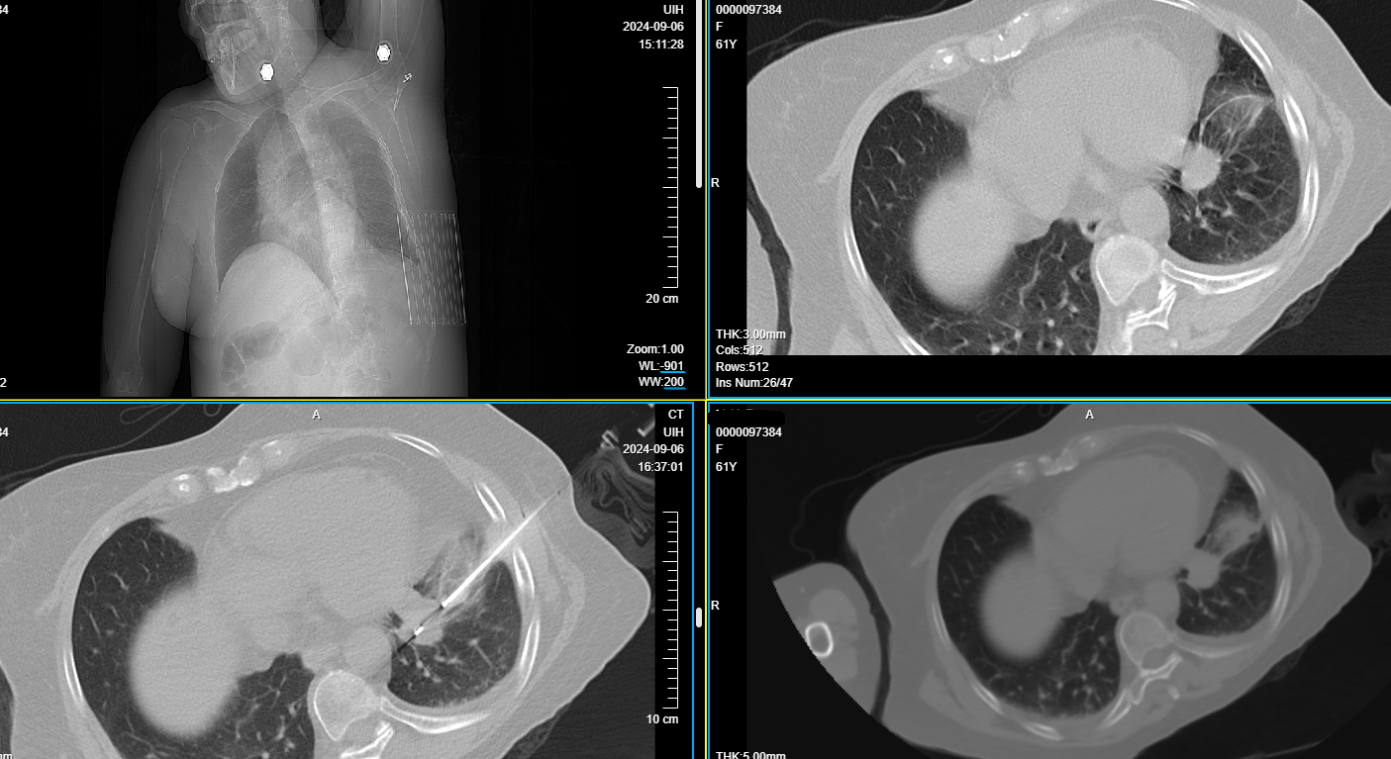

40.
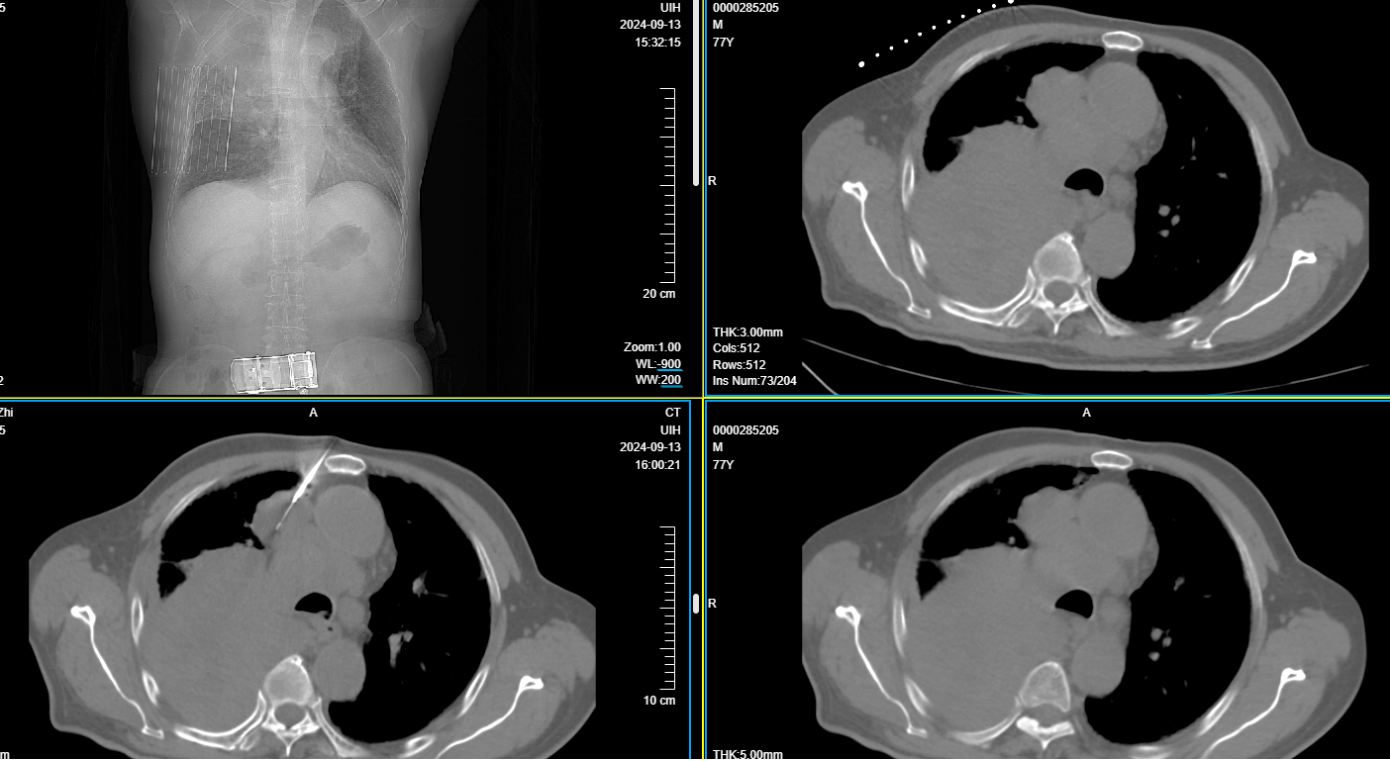

41.
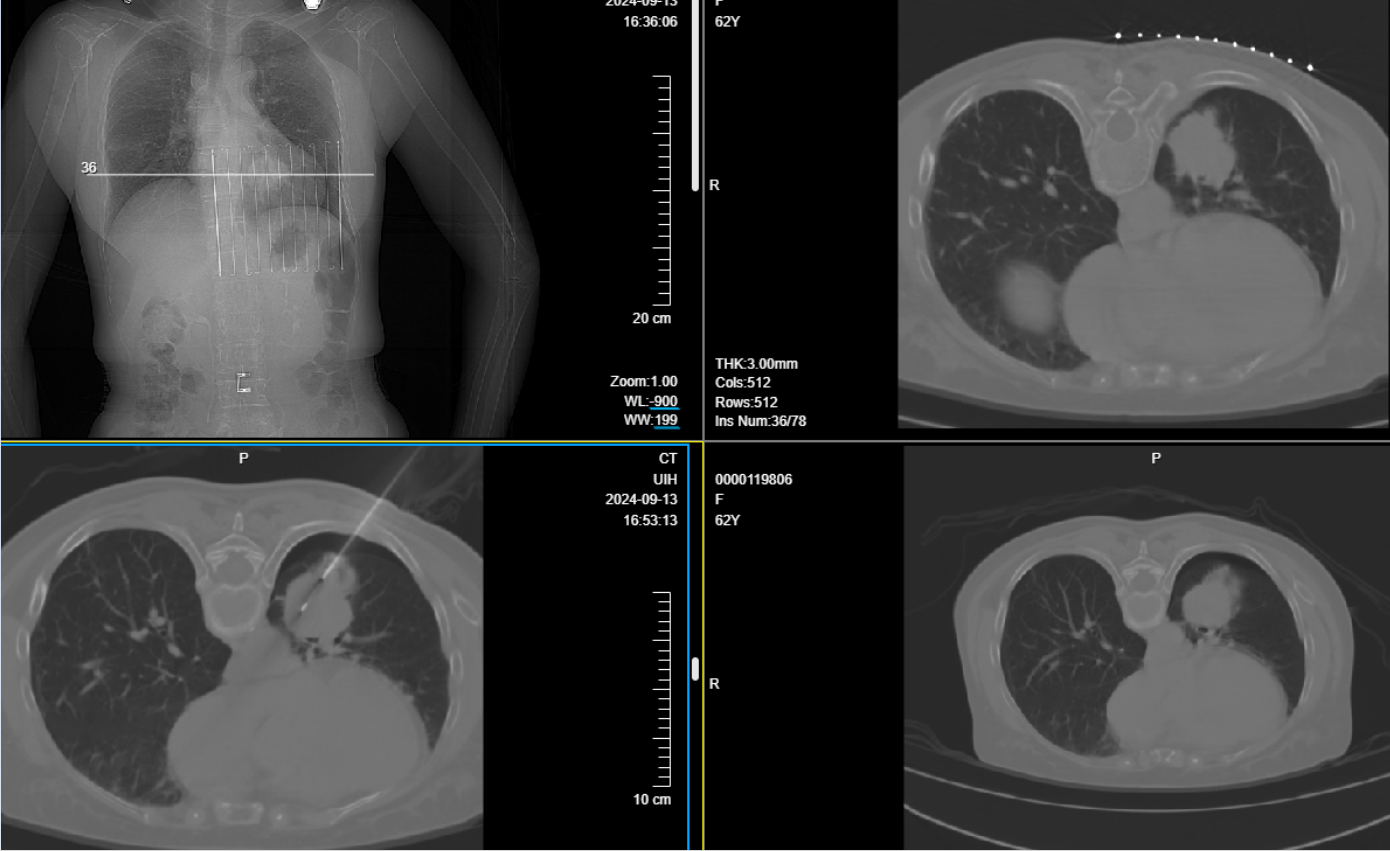

Supplement: Supplementary file 1 [file DataSheet1.docx]
